# MAP3K8/TPL-2/COT is a potential predictive marker for MEK inhibitor treatment in high-grade serous ovarian carcinomas

**DOI:** 10.1038/ncomms9583

**Published:** 2015-10-12

**Authors:** Tina Gruosso, Camille Garnier, Sophie Abelanet, Yann Kieffer, Vincent Lemesre, Dorine Bellanger, Ivan Bieche, Elisabetta Marangoni, Xavier Sastre-Garau, Virginie Mieulet, Fatima Mechta-Grigoriou

**Affiliations:** 1 Stress and Cancer Laboratory, Institut Curie, 26, rue d'Ulm, Paris 75248, France; 2 Inserm, Genetics and Biology of Cancers, U830, Paris F-75248, France; 3 Genomics and Biology of the Hereditary Breast Cancers, Institut Curie, 26, rue d'Ulm, Paris 75248, France; 4 Department of Pharmacogenomics, Institut Curie, 26, rue d'Ulm, Paris 75248, France; 5 Translational Research Department, Laboratory of Precinical Investigation, Institut Curie, 26, rue d'Ulm, Paris 75248, France; 6 Department of Pathology, Institut Curie, 26, rue d'Ulm, Paris 75248, France

## Abstract

Ovarian cancer is a silent disease with a poor prognosis that urgently requires new therapeutic strategies. In low-grade ovarian tumours, mutations in the MAP3K *BRAF* gene constitutively activate the downstream kinase MEK. Here we demonstrate that an additional MAP3K, MAP3K8 (TPL-2/COT), accumulates in high-grade serous ovarian carcinomas (HGSCs) and is a potential prognostic marker for these tumours. By combining analyses on HGSC patient cohorts, ovarian cancer cells and patient-derived xenografts, we demonstrate that MAP3K8 controls cancer cell proliferation and migration by regulating key players in G1/S transition and adhesion dynamics. In addition, we show that the MEK pathway is the main pathway involved in mediating MAP3K8 function, and that MAP3K8 exhibits a reliable predictive value for the effectiveness of MEK inhibitor treatment. Our data highlight key roles for MAP3K8 in HGSC and indicate that MEK inhibitors could be a useful treatment strategy, in combination with conventional chemotherapy, for this disease.

## Abstract (web summary)

The identification of pharmaceutically targetable biomarkers is essential for improving therapeutic options for cancer patients. In this study, the authors identify MAP3K8 as a potential predictive marker for MEK inhibitor treatment in patients with aggressive ovarian cancer.

## Graphical abstract

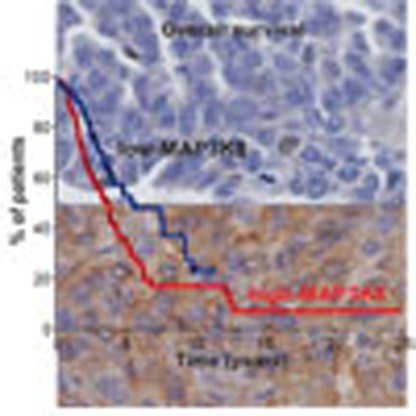

Epithelial ovarian cancers represent the fifth most frequent cause of cancer death in women. This silent disease is often diagnosed late, progresses rapidly and is thus associated with a poor prognosis. Although patients are initially quite sensitive to conventional platinum–taxane chemotherapy, most women relapse and ultimately die of the disease. This alarming observation highlights the urgent need to decipher ovarian tumours at a molecular level to develop more effective therapeutic strategies. To date, ovarian carcinomas have been mainly classified according to their histological subtype, grade and stage. Seventy percent of them are of the serous histological subtype, more than 75% of which are classified as high-grade tumours according to the two-tier MD Anderson Cancer Centre system^1^. Despite extensive studies related to the molecular characterization of high-grade serous ovarian carcinoma (HGSC) over the past few years^2^,^3^,^4^,^5^,^6^,^7^,^8^,^9^, new key players with therapeutic potential are still needed to be identified.

Low-grade and high-grade serous ovarian cancers exhibit distinct genetic alterations, molecular patterns and clinical behaviours^10^,^11^. *KRAS/BRAF* mutations are present in ∼70% of low-grade tumours, but only in 1% of those classified as high grade^4^,^12^. As BRAF is one of the two main mitogen-activated protein kinase kinase kinases (MAP3Ks) that regulate mitogen-activated extracellular signal-regulated kinase (MEK), *KRAS/BRAF* mutations result invariably in constitutive MEK activation. MEK inhibitors are thus of particular therapeutic interest for low-grade tumours harbouring *KRAS/BRAF* mutations^13^,^14^,^15^. MEK inhibitors have already been shown to be effective in *BRAF*-mutated melanoma patients who had developed resistance to anti-BRAF treatment^16^. Indeed, following treatment with BRAF inhibitors, some patients exhibit a chronic MEK activation and develop resistance through constitutive expression of the serine threonine kinase MAP3K8/TPL-2/COT, the other MAP3K upstream of MEK^14^,^17^. Similarly, we show here that a large proportion of HGSC exhibit MAP3K8 accumulation and subsequent MEK activation, independently of *KRAS/BRAF* mutation.

MAP3K8 controls several signalling pathways, including the MAPK pathway MEK/ERK in a cell-type- and stimulus-specific manner^18^. In the absence of any stimulus, MAP3K8 belongs to a ternary complex, comprising the nuclear factor-κB subunit precursor NF-κB1/p105, and the A20-binding inhibitor of NF-κB2 (ABIN-2), which inhibits its kinase activity^19^,^20^,^21^,^22^. Upon stimulation, MAP3K8 is released from this complex and is phosphorylated at multiple sites, two of which—threonine 290 (T290) and serine 400 (S400)—are required for full catalytic activity and subsequent MEK phosphorylation^23^,^24^,^25^. Recently, nutrient availability and a protein phosphatase 2A-dependent mechanism have also been shown to be required for MAP3K8 activation, thus revealing a new layer of complexity^26^. Despite increasing interest, MAP3K8 function in tumour development is still highly controversial^27^,^28^,^29^. MAP3K8 overexpression is observed in many human cancers—possibly because of genetic amplification^30^,^31^,^32^,^33^—but unlike *BRAF*^34^,^35^, *MAP3K8* somatic mutation is a rare event^27^,^36^,^37^,^38^,^39^.

Here, we provide new insights into the role of MAP3K8/TPL-2/COT in tumourigenesis and identify this kinase as a new biological prognostic marker with predictive value for MEK inhibitors in HGSC. We demonstrate that MAP3K8 pro-tumourigenic properties are mainly mediated by the MEK/ERK/p90RSK pathway. Furthermore, we identify key regulators of the G1/S transition and adhesion dynamics—namely cyclin D1 and focal adhesion kinase (FAK)—as MAP3K8 effectors. As there are no fully validated targetable molecular markers currently available for this pathology, our data indicate that MAP3K8/TPL-2/COT could be such a biomarker and define MEK inhibitors as a new promising therapeutic option for HGSC patients, in combination with conventional therapy.

## Results

### MAP3K8 accumulation is of poor prognosis in HGSC patients

**Figure 1:**
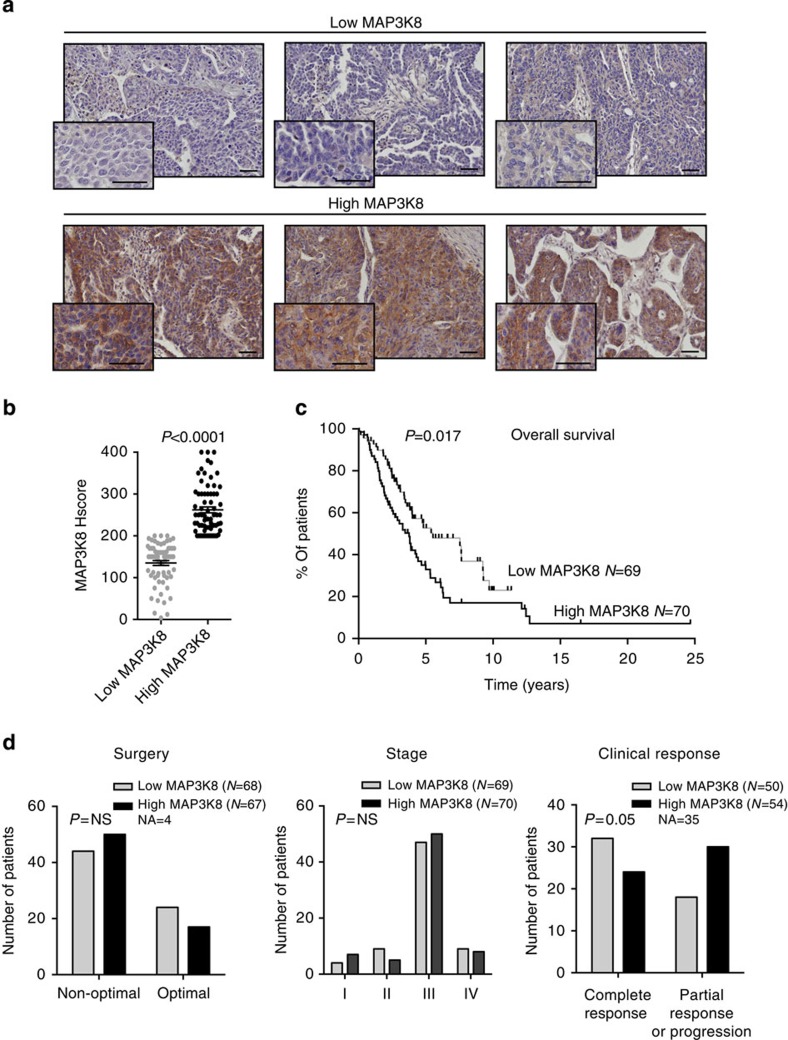
MAP3K8 is a prognostic marker for HGSC patients. (**a**) Representative views of MAP3K8 immunostaining from 139 human HGSC that exhibit low-MAP3K8 (top panel) or high-MAP3K8 (bottom panel) protein levels (see below). Scale bars, 50 μm. (**b**) Scatter plot of MAP3K8 histological score in the epithelial compartment (Hscore=staining intensity (0–4) × % of positive epithelial cells quantified from MAP3K8 immunostaining, as shown in **a**). Two subgroups of HGSC patients have been defined according to MAP3K8 Hscores, as low MAP3K8 (*N*=69 patients) or high MAP3K8 (*N*=70 patients) based on the median (=200). *P* value is based on the Student's *t*-test. Data are shown as mean±s.e.m. (**c**) Kaplan–Meier curves showing overall survival of HGSC patients, with respect to low-MAP3K8 (*N*=69 patients) or high-MAP3K8 (*N*=70 patients) protein levels. *P* value is based on the log-rank test. (**d**) Bar plots showing association of MAP3K8 protein levels with clinical parameters, when available, such as surgery (left panel), defined as optimal (*N*=41) or non-optimal (*N*=94), stage (middle panel) as established by clinicians (stage I, *N*=11; stage II, *N*=14; stage III, *N*=97; stage IV, *N*=17) and clinical response (right panel) considered as complete response (*N*=56) or partial response or progression (*N*=48). *P* values are based on Fisher's exact test.

There is clear evidence of compensatory mechanisms between the two main MAP3K, namely BRAF and MAP3K8, in regulating the MEK/ERK signalling pathway^17^. We thus investigated the role of MAP3K8 in human HGSC, in which *BRAF* mutations have been shown to be extremely rare^4^,^12^. We first tested the impact of MAP3K8 protein levels on patient survival (Fig. 1). Prior to any immunohistochemistry (IHC) analysis on human HGSC samples, we confirmed the specificity of our antibody for MAP3K8 protein by performing IHC on MAP3K8-depleted SKOV3 ovarian cancer cells in the same conditions as for the clinical samples (Supplementary Fig. 1A,B). Using this MAP3K8-specific antibody, we then performed IHC analysis on a large cohort of patients (see Supplementary Table 1 for clinical details and patient information), gathering 139 HGSC (Fig. 1a). Quantification of MAP3K8 histological staining in epithelial cells enabled us to identify two subgroups of patients characterized either by low or high MAP3K8 protein levels (Fig. 1a), using the median MAP3K8 histological score (Hscore) as a cut-off (Fig. 1b). Interestingly, MAP3K8 protein levels exhibited a prognostic value, as overall survival was markedly shortened in patients whose tumours exhibited high MAP3K8 protein levels (Fig. 1c). This MAP3K8 prognostic value only relates to protein and not messenger RNA (mRNA) levels, most probably because MAP3K8 mRNA and protein levels do not correlate in HGSC (Supplementary Fig. 1C). MAP3K8 prognostic value in HGSC could not be explained by surgery efficiency or tumour stage, as there was an equal distribution of advanced stage or partially resected tumours in the low- and high-MAP3K8 subgroups of patients (Fig. 1d, left and middle panels). In contrast, there was a significant association between MAP3K8 protein levels and clinical response to treatment, as patients exhibiting a partial response to treatment or a progressive disease preferentially harbour tumours with high MAP3K8 protein levels (Fig. 1d, right panel). The same tendency was observed, when considering either fully or partially resected tumours (Supplementary Fig. 1D), although the number of tumours for which we had access to all required information (debulking status, MAP3K8 protein level and response to treatment) was quite low in the Curie cohort. As MEK is activated in tumours in which MAP3K8 accumulates (see below), we confirmed the impact of MAP3K8/MEK on patient response to treatment in partially resected HGSC by analysing MEK phosphorylation in the The Cancer Genome Atlas (TCGA) database (Supplementary Fig. 1E). MAP3K8-mediated effect on patient response to treatment was independent of the BRCAness status, as there was an equal distribution of BRCAness and non-BRCAness patients^40^ in the high- versus low-MAP3K8 subgroup of HGSC Curie patients (*P*=0.2 by Fisher's exact test). Thus, our results indicate that in the absence of *BRAF* mutations, MAP3K8 protein is a new prognostic marker that might be an interesting therapeutic target in HGSC.

### MAP3K8 is pro-tumorigenic in ovarian cancer cells

**Figure 2:**
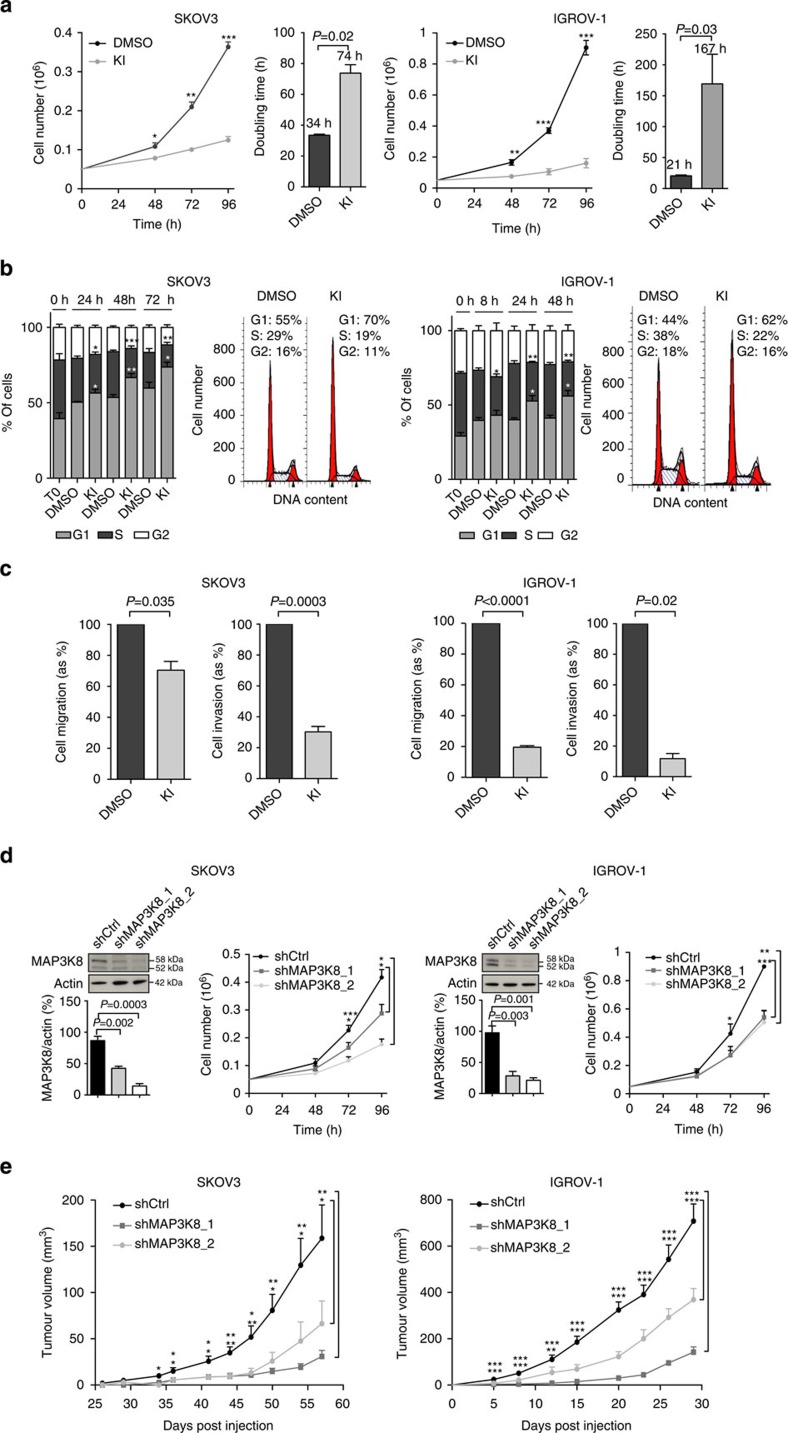
MAP3K8 controls proliferation, migration and invasion of ovarian cancer cells and tumour growth in mouse xenograft models. (**a**) Left: growth curves of SKOV3 and IGROV-1 ovarian cancer cell lines (OCCLs) treated either with MAP3K8 kinase inhibitor (KI) or with DMSO (vehicle medium for KI), for the indicated times (*n*=3). Right: bar plots show the doubling times of SKOV3 and IGROV-1 OCCL either untreated (DMSO) or treated with MAP3K8 kinase inhibitor (KI) (*n*=3). (**b**) Left: bar plots represent the percentage of SKOV3 and IGROV-1 OCCL in G1, S and G2 phases of the cell cycle, in control condition (DMSO) or following treatment with MAP3K8 KI, for the indicated times. For each cell line, the T0 time point represents the state of the cells just before any treatment (*n*=3). Right: representative cell-cycle distribution of SKOV3 and IGROV-1 OCCL without (DMSO) or with KI treatment. (**c**) Bar plots represent cell migration (left panels) or invasion (right panels) of SKOV3 and IGROV-1 OCCL either treated with MAP3K8 KI or with the vehicle medium for KI (DMSO) (*n*⩾3). (**d**) Left up: representative western blot showing MAP3K8 protein levels in stable cell lines expressing non-targeting shRNA (shCtrl) or two different MAP3K8-targeting shRNA (shMAP3K8_1 and shMAP3K8_2), derived from SKOV3 and IGROV-1 OCCL, as indicated. Actin is used as an internal control for protein loading. Left down: bar plot showing the ratio of MAP3K8/Actin protein levels, as assessed by densitometry analysis of western blots (as shown above) and expressed as percentage of shCtrl (*n*=3). Right, Growth curve of stable cell lines (shCtrl, shMAP3K8_1 and shMAP3K8_2) derived from SKOV3 and IGROV-1 OCCL, for the indicated times (*n*=3). (**e**) Tumour growth curves over time from one representative experiment of xenografted stable cell lines expressing shCtrl, shMAP3K8_1 or shMAP3K8_2 derived from SKOV3 and IGROV-1 OCCL (*N*⩾10 tumours per group). For all panels, data are shown as mean±s.e.m. *P* values are based on a one-sample *t*-test (**c**) and on the Student's *t*-test (**a**,**b**,**d**,**e**). *P≤0.05; **P≤0.005 and ***P≤0.0005. *n* stands for the number of replicated independent experiments.

IHC images from human HGSC samples show that MAP3K8 is highly expressed in epithelial cancer cells (Fig. 1a), which supports the possibility that this protein has a cell-autonomous function. To test this hypothesis, we analysed MAP3K8 functions in two independent human ovarian cancer cell lines (SKOV3 and IGROV-1), which exhibit high MAP3K8 protein levels (Supplementary Fig. 2A) and are known to be either null or inactivated for *TP53*, a common feature in almost all HGSC^12^,^41^,^42^. We first investigated the impact of MAP3K8 inhibition in these two cell lines using a specific ATP-competitive inhibitor of MAP3K8 (referred to hereafter as KI, Calbiochem #616373) (Fig. 2a–c), the specificity and activity of which have been previously characterized and tested in depth^43^,^44^,^45^. MAP3K8 inhibition by KI significantly reduced the total number of cells in the two ovarian cancer cell lines tested (Fig. 2a, left panels). Interestingly, we observed an increase in cell-doubling time following KI treatment (Fig. 2a, right panels), without any impact on cell viability (Supplementary Fig. 3A). These results suggest that MAP3K8 inhibition might affect cell-cycle progression rather than cell death. We thus performed cell-cycle analysis using flow cytometry and observed a significant increase in the percentage of cells in G1 phase compared to untreated control cells (Fig. 2b). Cells therefore tend to accumulate in G1 after MAP3K8 inhibition, which reduces their proliferation rate. To further determine whether MAP3K8 might also affect ovarian tumour cell migration and/or invasion, we performed transwell assay experiments. Upon KI treatment, MAP3K8 inhibition significantly reduced both cell migration (Fig. 2c, left panels) and cell invasion (Fig. 2c, right panels), in the two human ovarian cancer cell lines tested. Moreover, we validated these observations in ovarian cancer cell lines derived from mouse ovarian surface epithelial cells (MOSECs)^46^, in which we also observed a decrease in cell proliferation and cell migration upon MAP3K8 inhibition (Supplementary Fig. 3B,C). These results highlight that the cell-autonomous functions of MAP3K8 during ovarian tumourigenesis occur in various cell lines and are conserved among species. We used a second strategy to confirm the role of MAP3K8 in ovarian cancer cells and tumour development—KI could not be tested *in vivo* due to expensive cost established by the manufacturer. We stably inactivated MAP3K8 in SKOV3 and IGROV-1 cell lines by expressing short hairpin RNAs (shRNAs) that were either non-targeting (shCtrl) or targeting *MAP3K8* (shMAP3K8_1 and shMAP3K8_2) (Fig. 2d,e). We observed a significant knockdown of MAP3K8 protein in both shMAP3K8_1 and shMAP3K8_2 stable cell lines (Fig. 2d, left panels; for the original blots, please see Supplementary Fig. 8A), although we never reached a complete inhibition of MAP3K8, most probably because this would be toxic for ovarian cancer cells. Still, this is clinically relevant as it mimics what we observed in patients with HGSC, with MAP3K8 level being either low or high but not null (as shown in Fig. 1a,b). On the basis of western blot analyses, we confirmed that SKOV3 and IGROV-1 cell lines belong to the high-MAP3K8 subgroup, while shMAP3K8_1 and shMAP3K8_2 stable cell lines derived from parental SKOV3 and IGROV-1 belong to the low-MAP3K8 subgroup (Supplementary Fig. 2B,C). Indeed, the median value of MAP3K8 protein level separating low- and high-MAP3K8 HGSC tumour samples (as assessed by densitometry analyses of western blots) was equal to 0.9 (Supplementary Fig. 2B and corresponding legend for detailed explanations). The mean values obtained for parental SKOV3 and IGROV-1 cell lines were above 0.9 while they are below that threshold for shMAP3K8_1 and shMAP3K8_2 stable cell lines derived from SKOV3 and IGROV-1 cells (Supplementary Fig. 2C), arguing that the parental cell lines belong to the high-MAP3K8 subgroup, while shMAP3K8-derived cells belong to the low-MAP3K8 subgroup. As observed upon KI treatment, we confirmed that MAP3K8 stable silencing significantly reduced cell proliferation in all ovarian cancer cell lines tested (Fig. 2d, right panels), without affecting their viability (Supplementary Fig. 3D). Finally, using mouse xenograft models, we observed that tumour growth was severely impaired following MAP3K8 depletion compared with control tumours, result validated in shMAP3K8_1 and -2 stable cell lines derived from the two independent ovarian cancer cell lines tested (Fig. 2e). The IGROV-1 cell line was the only one, which generated lung metastases. Using this model, we observed that the number of mice with lung metastases was significantly lower following MAP3K8 depletion (Supplementary Fig. 3E). Thus, our data indicate that MAP3K8 inhibition significantly reduces ovarian cancer cell proliferation, migration and invasion *in vitro*, as well as tumour growth *in vivo*. These observations strongly argue in favour of a cell-autonomous function for MAP3K8 in ovarian tumours. Taken together, our data show that MAP3K8 regulates key features of ovarian cancer cells and promotes tumourigenesis *in vivo*, which explains, at least in part, that the high level of MAP3K8 protein is associated with a poor prognosis in human HGSC.

### MEK is activated downstream MAP3K8 in ovarian cancer cells

**Figure 3:**
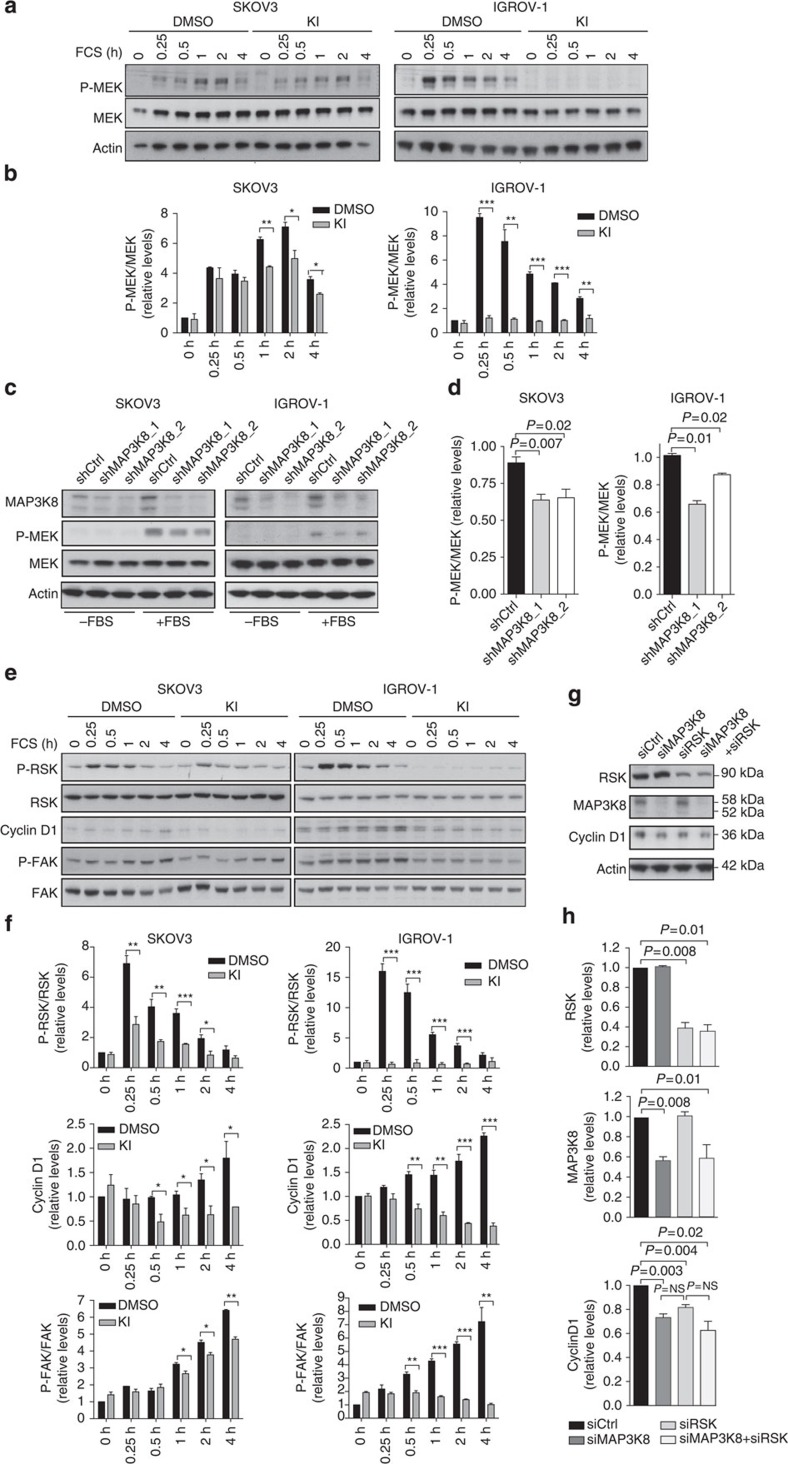
MAP3K8 is upstream of MEK and involved in regulating cyclin D1 and FAK in ovarian cancer cells. (**a**) Representative western blots showing the phosphorylated form of MEK (P-MEK) and total MEK protein levels in SKOV3 and IGROV-1 OCCL either treated with MAP3K8 kinase inhibitor (KI) or with KI vehicle (DMSO), for 1 h before serum (FBS) stimulation for the indicated times. Actin is used as an internal control for protein loading. (**b**) Bar plots showing P-MEK/MEK ratio, as assessed by densitometry analysis of western blots (as shown in **a**) and normalized to the DMSO *t*=0 h time point (*n*=3). (**c**) Western blots showing MAP3K8, P-MEK and MEK protein levels in stable cell lines (shCtrl, shMAP3K8_1 and shMAP3K8_2) derived from SKOV3 and IGROV-1 OCCL and kept without serum (−FBS) or stimulated with serum (+FBS). Actin is used as an internal control for protein loading. (**d**) Bar graphs showing P-MEK/MEK ratio assessed by densitometry analysis of western blots (as shown in **c**) and normalized to shCtrl (*n*⩾3). (**e**) Representative western blots showing P-p90RSK, p90RSK, cyclin D1, P-FAK and FAK protein levels upon serum (FBS) stimulation for the indicated times in SKOV3 and IGROV-1 OCCL pretreated either with MAP3K8 KI or with KI vehicle (DMSO). Actin is used as an internal control for protein loading. (**f**) Bar plots showing P-p90RSK/p90RSK ratio, cyclin D1 and P-FAK/FAK ratio, as assessed by densitometry analysis of western blots (as shown in **e**) and expressed as fold change compared with the DMSO *t*=0 h time point (*n*⩾3). (**g**) Representative western blots showing P-p90RSK, MAP3K8 and cyclin D1 upon silencing of either p90RSK or MAP3K8, or following combined inactivation of both MAP3K8 and p90RSK in SKOV3 ovarian cancer cells. Actin is used as an internal control for protein loading. (**h**) Bar plots showing p90RSK, MAP3K8 and cyclin D1 protein levels, as assessed by densitometry analysis of western blots (as shown in **e**) and expressed as fold change compared with the control (siCtrl) (*n*=3). For all panels, data are shown as mean±s.e.m. *P* values are based on the Student's *t*-test. *P≤0.05; **P≤0.005 and ***P≤0.0005. *n* stands for the number of replicated independent experiments.

MAP3K8 downstream signalling is stimulus- and cell-type specific^18^,^39^,^47^. The MAPK pathways MEK/ERK, JNK and p38MAPK as well as the NF-κB p65 pathway can be directly activated by MAP3K8 (refs 48, 49, 50, 51). We thus investigated the impact of MAP3K8 inhibition on the above-mentioned pathways in ovarian cancer cells (Fig. 3). Serum-induced MEK phosphorylation was significantly reduced upon MAP3K8 inhibition, either by KI treatment (Fig. 3a,b; for the original blots, please see Supplementary Fig. 8B) or by silencing (Fig. 3c,d). In contrast, MAP3K8 inhibition either mediated by KI treatment (Supplementary Fig. 4A,B) or MAP3K8 silencing (Supplementary Fig. 5A,B) did not reproducibly affect the serum-induced JNK, p38MAPK and NF-κB p65 phosphorylation in these cell lines. Thus, MEK was the only common pathway downregulated upon MAP3K8 inhibition using either of the two complementary strategies in the two independent ovarian cancer cell lines tested. Therefore, MAP3K8 functions are most reliably mediated through MEK in ovarian cancer cells.

We next aimed at investigating MAP3K8 downstream effectors that are involved in controlling cell-cycle progression and migration. We examined the role of MAP3K8 on p90RSK, which functions at the crossroads of MEK signalling in cell-cycle progression and migration^52^,^53^,^54^. We observed a significant decrease in p90RSK phosphorylation upon MAP3K8 inhibition in the two ovarian cancer cell lines tested (Fig. 3e,f; for the original blots, please see Supplementary Fig. 8B). In many cell types, the p90RSK activation downstream MEK pathway is required for the G1/S transition by inducing cyclin D1 expression^55^. In agreement with the accumulation of ovarian cancer cells in G1 observed upon KI treatment (Fig. 2), we noticed a significant and reproducible decrease in cyclin D1 protein levels in the different ovarian cancer cell lines tested (Fig. 3e,f; for the original blots, please see Supplementary Fig. 8B). In addition, another substrate of MEK/p90RSK pathway known to regulate cell migration is the Focal Adhesion Kinase (FAK)^56^,^57^. FAK is a non-receptor tyrosine kinase involved in the regulation of adhesion dynamics that is phosphorylated at multiple sites, including the serine 910 (S910). We confirmed that FAK phosphorylation on S910 residue was repeatedly impaired in the different KI-treated ovarian cancer cell lines analysed (Fig. 3e,f; for the original blots, please see Supplementary Fig. 8B), consistent with the reduced cell migration we observed upon MAP3K8 inhibition (Fig. 2c). Taken as a whole, our data strongly support that p90RSK could mediate MAP3K8-dependent control of cell proliferation and migration, acting on cyclin D1 and FAK, well-known regulators of the G1/S checkpoint and adhesion dynamics, respectively. Finally, to fully demonstrate p90RSK was the key effector mediating MAP3K8/MEK actions in ovarian cancer cells, we inactivated it alone or in combination with MAP3K8 silencing (Fig. 3g,h). First, we observed that inactivating either of the two protein kinases was sufficient to reduce cyclin D1 protein levels in ovarian cancer cells, confirming that both MAP3K8 and p90RSK were required for cyclin D1 expression. Moreover, the combined inhibition of both MAP3K8 and p90RSK did not show any additive effect, suggesting that p90RSK is the only key player downstream of MAP3K8, involved in regulating cyclin D1 in ovarian cancer cells. Taken as a whole, our data indicate that p90RSK, downstream of MEK/ERK, could mediate MAP3K8-dependent control of cell proliferation by acting on cyclin D1, a well-known regulator of the G1/S checkpoint.

### MAP3K8 is a significant readout for MEK signalling in HGSC

**Figure 4:**
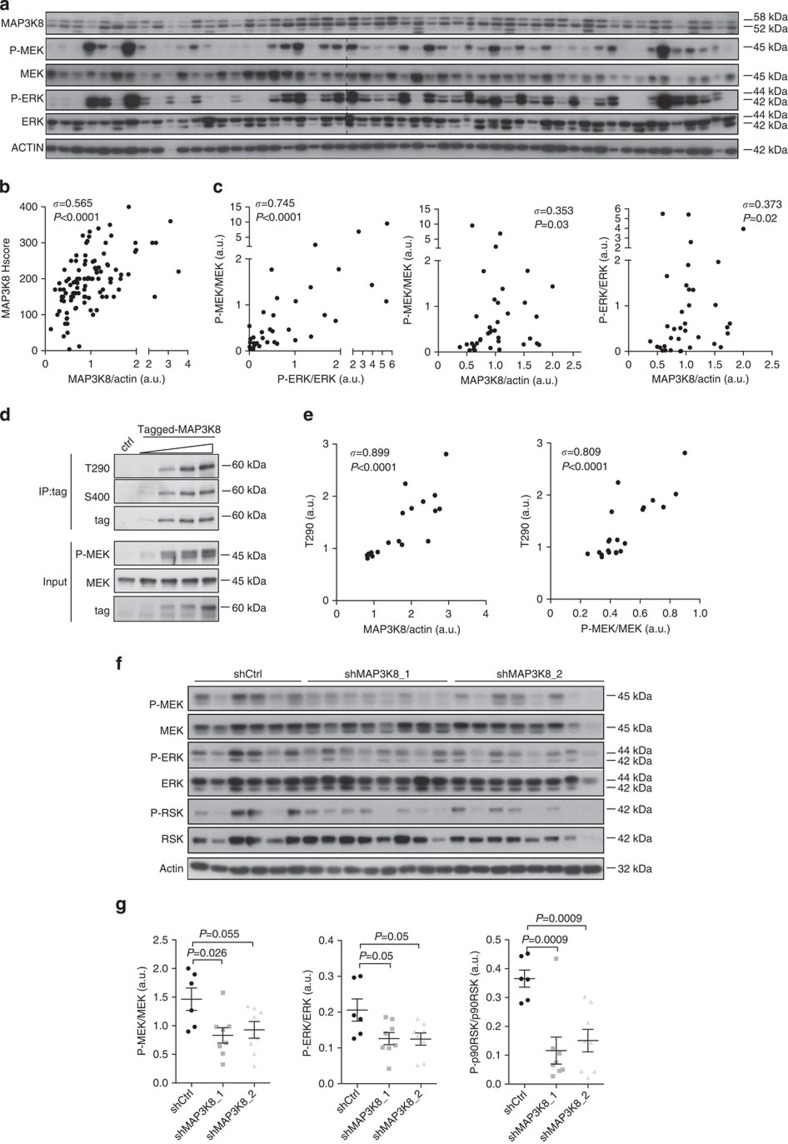
MAP3K8 protein level correlates with its kinase activity and MEK activation in human HGSC. (**a**) Representative western blots showing MAP3K8, P-MEK, MEK, P-ERK and ERK protein levels from human HGSC protein extracts enriched in epithelial cells. On a total number of 108 HGSC analysed, representative results on 54 patients are shown. Actin is used as an internal control for protein loading. (**b**) Correlation plot between MAP3K8 protein levels evaluated by western blots (as shown in **a**) and histological scoring in epithelial cells (as shown in Fig. 1a,b) from 108 HGSC tumour samples. (**c**) Correlation plots between P-MEK/MEK and P-ERK/ERK (left); MAP3K8/actin and P-MEK/MEK (middle); MAP3K8/actin and P-ERK/ERK (right) in human HGSC. Values have been assessed by densitometry analysis of western blots (as those shown in **a**). (**d**) Western blots showing MAP3K8 phosphorylation on threonine 290 (T290) or on serine 400 (S400), as well as P-MEK and MEK protein, before (input) or after Myc-Tag-specific antibody immunoprecipitation (IP:tag) from protein extracts of SKOV3 cells transfected with empty vector (Ctrl) or increasing amounts of myc-tagged MAP3K8 expression vector (tagged MAP3K8). The Myc-tag is used as an internal control for MAP3K8 protein levels (tag). (**e**) Correlation plot between P-MAP3K8 (T290) and MAP3K8 protein levels (left panel) and between P-MAP3K8 (T290) protein levels and P-MEK/MEK ratio (right panel), as assessed by densitometry analysis of western blots (as shown in **d**) (*n*=4). (**f**) Western blots showing P-MEK, MEK, P-ERK, ERK, P-p90RSK and p90RSK protein levels in mouse xenografted tumours (*N*⩾6 tumours per group) derived from shCtrl, shMAP3K8_1 or shMAP3K8_2 SKOV3 stable cell lines (tumour growth described above, Fig. 2e). Actin is used as an internal control for protein loading. (**g**) Scatter plot of P-MEK/MEK (left), P-ERK/ERK (middle) and P-p90RSK/p90RSK (right) ratios in xenograft tumours derived from shCtrl, shMAP3K8_1 and shMAP3K8_2 SKOV3 stable cell lines, as assessed by densitometry analysis of the western blots shown in **f**. *P* values are based on the Student's *t*-test. Data are shown as mean±s.e.m. (*N*⩾6 tumours per group). For all correlation plots, correlation coefficients *σ* and *P* values are based on Spearman's rank correlation test. *n* stands for the number of replicated independent experiments. a.u., arbitrary unit.

MAP3K8 has been defined as an important upstream regulator of MEK in some cancers^17^,^45^,^58^. As we observed that MEK signalling is the main pathway activated downstream of MAP3K8 in ovarian cancer cells, we investigated the role of the MAP3K8/MEK/ERK pathway in HGSC patients. We first tested whether MAP3K8 protein accumulation in HGSC correlated with MEK activation (Fig. 4). We assessed MAP3K8 protein levels, as well as MEK and ERK activation, using western blot analysis on a large set of HGSC samples, enriched in epithelial cancer cells, up to 73% on average (Fig. 4a; for the original blots, please see Supplementary Fig. 8C). This was our only option to measure P-MEK/MEK ratio according to MAP3K8 protein levels, IHC analysis of P-MEK being unavailable. Similar to what we observed using IHC staining (Fig. 1a), western blot analysis confirmed that MAP3K8 was differentially expressed among human HGSC (Fig. 4a). Importantly, there was a significant correlation between MAP3K8 protein levels evaluated either by western blots from human HGSC protein extracts enriched in epithelial cells or by IHC scoring of MAP3K8 staining in epithelial cells (Fig. 4b). For each type of experiments (IHC and western blots), the low- and high-MAP3K8 subgroups of HGSC tumours have been defined below and above the median value, respectively. As expected, we showed a significant positive correlation between MEK and ERK activation in HGSC tumours (Fig. 4c, left panel). Importantly, we observed MAP3K8 protein levels correlated with both MEK (Fig. 4c, middle panel) and ERK (Fig. 4c, right panel) activation in HGSC, suggesting a predominant effect of MAP3K8 on the MEK/ERK pathway in this pathology. We then investigated whether MAP3K8 differential accumulation in HGSC could potentially be due to its association with NF-κB1/p105 and ABIN-2, which both regulate its stability^21^,^51^ (Supplementary Fig. 6). We indeed observed a positive correlation between MAP3K8 protein levels and levels of the components of the ternary complex to which it belongs (Supplementary Fig. 6A–D). This suggests that MAP3K8 differential expression in HGSC could be partly due to the regulation of its stability.

There is no existing tool for evaluating endogenous MAP3K8 kinase activity in tumours, the phosphorylated form of MAP3K8 being undetectable in protein extracts from tumours. To further demonstrate that the level of MAP3K8 protein correlates with its kinase activity and subsequent MEK/ERK activation, we analysed the MAP3K8 phosphorylation state in SKOV3 cells expressing increasing levels of MAP3K8 (Fig. 4d). Full catalytic activity of MAP3K8 requires its phosphorylation at both T290 and S400 (refs 23, 24, 25, 26). Increasing MAP3K8 expression levels in SKOV3 cells was sufficient to induce MAP3K8 phosphorylation at both phosphorylation sites and subsequent MEK activation, in a dose-dependent manner (Fig. 4d). Indeed, we observed a clear correlation between the MAP3K8 protein level and its phosphorylation state at the T290 and S400 phosphorylation sites (Fig. 4e, left panel; Supplementary Fig. 6E, left panel), indicating that the protein level and kinase activity of MAP3K8 are correlated in SKOV3 ovarian cancer cells. Interestingly, MAP3K8 phosphorylation state also correlated with MEK activation (Fig. 4e, right panel; Supplementary Fig. 6E, right panel), further highlighting that MAP3K8 kinase could be a significant readout for MEK activation in ovarian cancers. Finally, in agreement with this observation, we showed MEK activation and subsequent ERK and p90RSK phosphorylation were significantly impaired in mouse ovarian tumours following MAP3K8 depletion (Fig. 4f,g), further indicating that MAP3K8-dependent tumour growth is mediated by MEK/ERK/p90RSK activation *in vivo*. Taken together, our data show that MAP3K8 differentially accumulates in ovarian tumours along with the ternary complex known to regulate its stability. Importantly, MAP3K8 protein levels correlate with its kinase activity and subsequent MEK/ERK/p90RSK activation, which participates in ovarian tumour growth.

We next asked whether MAP3K8 accumulation was the only upstream kinase regulating MEK in HGSC patients. The other well-known MAP3 kinase that activates the MEK/ERK pathway is BRAF and its corresponding gene is often mutated in cancer, including low-grade HGSC^35^,^59^. However, as previously mentioned, using publicly available data sets from a large cohort of HGSC generated by TCGA^4^, we confirmed that the percentage of cells that contained the *BRAF* mutation was no more than 1%. Nevertheless, in 11% of cases we detected genetic amplification of the *BRAF* gene, but this did not correlate with MEK phosphorylation assessed by reverse-phase protein array (Supplementary Fig. 6F). It is thus unlikely that BRAF is involved in MEK/ERK pathway activation in HGSC. Therefore, these results strongly suggest that MAP3K8 might be the sole MAP3 kinase involved in MEK activation in HGSC. This hypothesis is supported by our data revealing that MAP3K8 protein levels correlate with activation of MEK pathway in ovarian cancer cells, mouse tumour models and human HGSC.

### MAP3K8 is a predictive marker for MEK inhibitor efficiency

**Figure 5:**
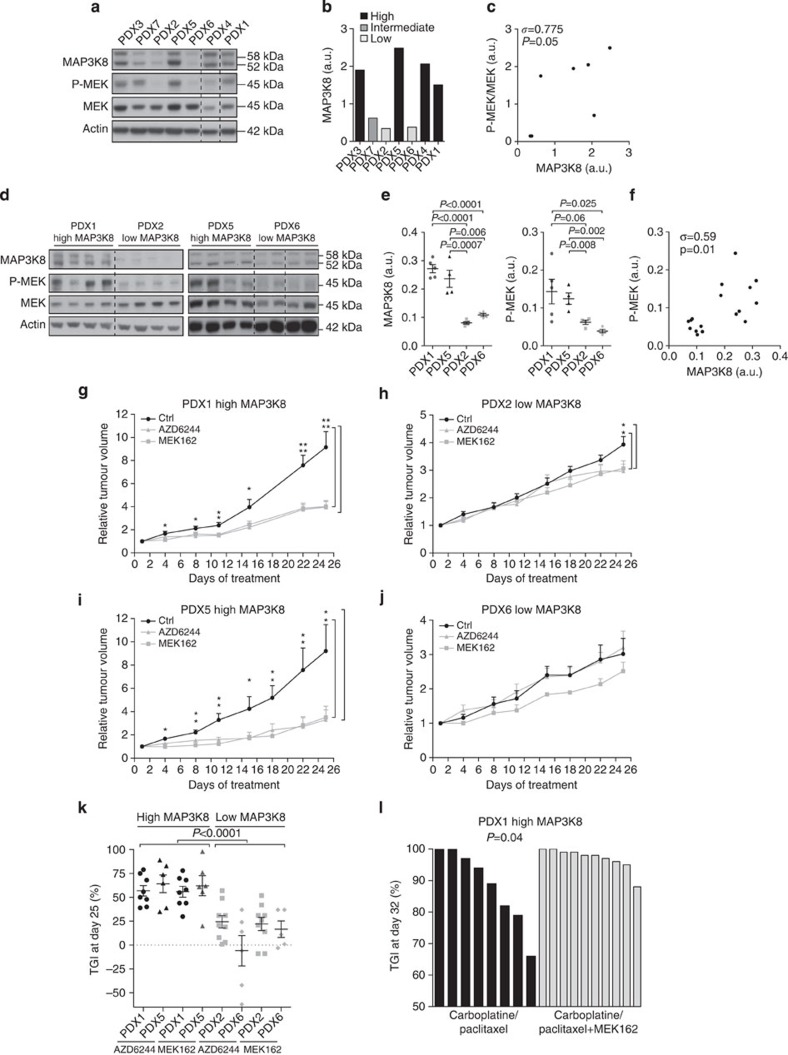
MAP3K8 is a predictive marker for MEK inhibitor treatments. (**a**) Western blots showing MAP3K8, P-MEK and MEK protein levels in seven different mouse models of patient-derived xenograft (PDX). One representative tumour is shown for each model. Actin is used as an internal control for protein loading. (**b**) Scatter plots of MAP3K8 protein levels in tumours from the seven PDX mouse models studied, as assessed by densitometry analysis of western blots. PDX models with high MAP3K8 protein levels (PDX1,3,4,5) are in black, while PDX models with low- or intermediate-MAP3K8 protein levels (PDX2,6,7) are in light or dark grey, respectively. (**c**) Correlation plot between P-MEK/MEK and MAP3K8 protein levels in tumours from the seven PDX models. (**d**) Western blots showing MAP3K8, P-MEK and MEK protein levels in tumours derived from PDX1,5 and PDX2,6 with high- and low-MAP3K8 protein levels, respectively. Actin is used as an internal control for protein loading. (**e**) Scatter plots of MAP3K8 (left) and P-MEK (right) protein levels, as assessed by densitometry analysis of the western blots shown in **a** in PDX with high- (PDX1,5, black) or low- (PDX2,6, grey) MAP3K8 protein levels (*N*⩾4 tumours per group). (**f**) Correlation between P-MEK and MAP3K8 protein levels in tumours derived from PDX1,2,5,6 models. (**g**,**h**,**i**,**j**) Relative tumour volume over time of PDX models exhibiting either high- (PDX1,5) (**g**,**i**) or low- (PDX2,6) (**h**,**j**) MAP3K8 protein levels. Mice were either untreated (Ctrl, black lines) or subjected to *per os* treatment with MEK inhibitors, including AZD6244 and MEK162 (grey lines) (*N*⩾6 mice per group). (**k**) Scatter plot of tumour growth inhibition (TGI) in AZD6244- and MEK162-treated groups, as compared with the control group in high- (PDX1,PDX5) or low- (PDX2,PDX6) MAP3K8 PDX models (*N*⩾6 tumours per group). (**l**) Bar plot of TGI per mouse in PDX1 (high-MAP3K8 model) following conventional therapy (carboplatin+paclitaxel) alone or in combination with MEK inhibitor (carboplatin+paclitaxel+MEK162) (*N*⩾8 mice per group). In **c** and **f**, correlation coefficients *σ* and *P* values are based on Spearman's rank correlation test. In **e**,**g** and **h**–**l**, *P* values are based on the Student's *t*-test. Data are shown as mean±s.e.m. *P≤0.05 and **P≤0.005. a.u., arbitrary unit.

As shown above, MEK is constitutively active in HGSC with high MAP3K8 protein levels, which suggests that inhibiting the MAP3K8/MEK pathway could be beneficial for these patients. Inhibiting MAP3K8 *in vivo* using KI was not possible due to cost reasons. Thus, we opted for inhibition of its direct substrate MEK, using two different ATP non-competitive MEK inhibitors, AZD6244/Selumetinib and MEK162, which have both been included in clinical trials for the treatment of low-grade serous ovarian cancer patients^15^,^60^. We tested our hypothesis using these MEK inhibitors on HGSC patient-derived xenografts (PDXs) (Fig. 5; Supplementary Fig. 7). First, we confirmed the reliability of these PDX models, showing that they exhibited the same histological features as the human primary tumours from which they derived (Supplementary Fig. 7A). As the subcutaneous route allows a more accurate measurement of tumour volume and drug treatment efficacy than the orthotopic route, we favoured this strategy, a choice that has also been validated in a recent study^61^. We identified seven PDX models exhibiting either low or high MAP3K8 protein levels (Fig. 5a,b; for the original blots, please see Supplementary Fig. 8D). Similar to human HGSC, we observed a significant correlation between MAP3K8 protein levels and MEK activation in the seven PDX models studied (Fig. 5c). Moreover, we validated that, within each PDX mouse model, the different tumours exhibit comparable MAP3K8 protein levels (Fig. 5d,e; for the original blots, please see Supplementary Figs 7B,C and 8E). Furthermore, we confirmed a greater MEK activation in the different tumours developed in a high-MAP3K8 PDX model (PDX1) compared with tumours from a low-MAP3K8 PDX model (PDX2) (Fig. 5d,e right panel), with a positive correlation between MAP3K8 protein level and MEK activation (Fig. 5f). Confirming the potential interest of MEK inhibitors for the treatment of HGSC patients with high MAP3K8 protein levels, we observed that both MEK inhibitors markedly reduced tumour growth in high-MAP3K8 PDX models (Fig. 5g,i; Supplementary Fig. 7D,E), compared with low- or intermediate-MAP3K8 PDX models (Fig. 5h,j; Supplementary Fig. 7F). Indeed, tumour growth inhibition was significantly higher in high-MAP3K8 PDX (60% in average) than in low-MAP3K8 PDX (16% in average) (Fig. 5k). Moreover, treatment with MEK inhibitors significantly reduced lung metastatic incidence in high-MAP3K8 PDX compared with low-MAP3K8 PDX (Supplementary Fig. 7G). As we observed a significant association between MAP3K8 protein levels and patient's response to treatment (Fig. 1d, right panel), we first validated that observation regarding P-MEK in the TCGA cohort of HGSC patients (http://cancergenome.nih.gov/; P-MEK status available in reverse-phase protein array data for 412 tumour samples, as well as primary therapy outcome, as defined in TCGA). We observed a significant association between P-MEK status and the primary therapy outcome (*P*=0.03 using Fisher's exact test). As observed in the Curie cohort of HGSC, this effect was not related to BRCAness, as there is no enrichment of BRCAness/non-BRCAness patients in low-/high-P-MEK subgroups (*P*=0.9 by Fisher's exact test). Taken as a whole, these data indicate that MAP3K8 accumulation and subsequent MEK pathway activation significantly reduces the efficiency of conventional therapies. We thus tested, on a PDX model exhibiting high MAP3K8 protein levels (PDX1), the impact of MEK inhibitor MEK162 in combination with carboplatine and taxanes, conventional therapies given to HGSC patients. We observed a significant increase in the number of complete tumour regression when MEK inhibitor is given in combination with carboplatine and taxanes, compared with conventional therapies alone (Fig. 5l). These data confirm that anti-MEK treatments could be beneficial, in addition to chemotherapy, for patients suffering from HGSC with high MAP3K8 protein levels. Taken as a whole, our data demonstrate the potential interest of MEK inhibitors as a new therapeutic strategy in ovarian tumourigenesis and identify MAP3K8 as a new predictive marker for the effectiveness of MEK inhibitors in human HGSC.

## Discussion

**Figure 6:**
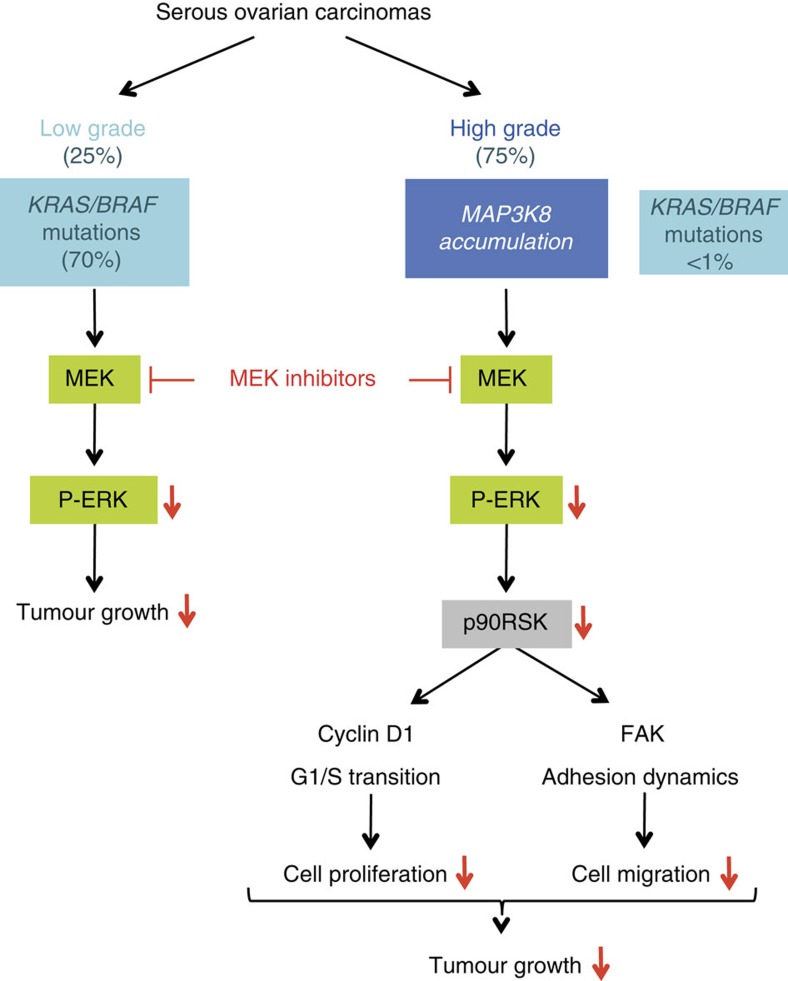
MAP3K8 exhibits pro-tumorigenic properties in HGSC and is a predictive marker for treatment with MEK inhibitor. Among epithelial ovarian cancers, the serous histological subtype is the most abundant and is subdivided into two subgroups according to grade (low versus high grade). Seventy percent cases of low-grade ovarian carcinomas exhibited *KRAS* or *BRAF* mutations and subsequent MEK activation, which led to the use of MEK inhibitors as a new line of treatment in recent clinical trials with some relevant efficiency. In contrast, *KRAS* or *BRAF* mutations remain extremely rare in HGSC (less than 1%). Yet, we demonstrate here that the MEK pathway is constitutively activated in half cases of HGSC. MAP3K8 (also referred as TPL-2 or COT) accumulation in HGSC mediates this constitutive MEK/ERK pathway activation, MAP3K8 being the other MEK kinase, besides RAF. MAP3K8 contributes to tumour growth via p90RSK-dependent regulation of cyclin D1 and FAK, key regulators of the G1/S transition and adhesion dynamics, respectively. Therefore, our study demonstrates for the first time that the use of MEK inhibitors should not be restricted to low-grade ovarian carcinomas, as they are currently in clinical trials, but could be extended to patients suffering from HGSC characterized by MAP3K8 accumulation.

Deciphering HGSC molecular patterns is crucial not only for understanding the pathogenesis of this heterogeneous disease but also for improving therapeutic options. The identification and characterization of targetable molecular markers is thus essential, especially for patients resistant to platinum regimen. Despite the molecular characterization of HGSC^2^,^3^,^4^,^5^,^6^,^7^ and the recent development of anti-poly ADP ribose polymerase and anti-angiogenic therapies^5^,^62^, no clear ‘druggable' target has yet been identified. Our work reveals that the MEK kinase MAP3K8/TPL-2/COT could be such a valuable biomarker. Indeed, HGSC with high MAP3K8 protein levels have a poor prognosis. Moreover, MAP3K8 is a key player in ovarian tumourigenesis by controlling progression through the cell cycle and the invasive properties of ovarian cancer cells. Finally, MEK/ERK is the major signalling pathway downstream of MAP3K8 that mediates these cell-autonomous functions. Consistent with these findings, MAP3K8 is a predictive marker for the efficiency of MEK inhibitors, which seem to be a promising new therapeutic alternative for HGSC patients with high-grade serous tumours characterized by MAP3K8 protein accumulation (see the model in Fig. 6).

MAP3K8 function in tumour development is highly controversial^32^,^45^,^63^,^64^. A major issue is whether MAP3K8 has deleterious or beneficial effects on tumour growth and cancer patient outcome. Our study addresses this issue in ovarian tumourigenesis. Indeed, we clearly demonstrate that high MAP3K8 protein levels are associated with poor prognosis in HGSC, indicating that MAP3K8 favours ovarian tumour development. Using shRNA targeting MAP3K8 or MAP3K8 kinase inhibitor, we have demonstrated for the first time that MAP3K8 positively controls ovarian cancer cell proliferation, migration and invasion, as well as tumour growth in mouse models. Moreover, we found that p90RSK, a main effector of MEK/ERK signalling, could mediate MAP3K8 cell-autonomous functions in ovarian cancer (Fig. 6). Indeed, MAP3K8 effectors, cyclin D1 and FAK, are key molecules involved in the G1/S transition and adhesion dynamics, respectively. This is consistent with the previously reported mechanism of MEK/ERK/p90RSK pathway regulation of cell-cycle progression and migration^52^,^53^,^54^,^55^, further indicating that MEK mediates MAP3K8 effects on the proliferation and motility of ovarian cancer cells. To our knowledge, this is the first time that the interplay between MAP3K8 and RSK in regulating cyclin D1 expression is shown in ovarian cancer cells and that RSK is the only key player downstream of MAP3K8 involved in this process. Importantly, MAP3K8 has been shown to play a key function in innate immune response. Indeed, the generation of *MAP3K8*^*−/−*^ mice clearly demonstrated MAP3K8 is required for production of the pro-inflammatory cytokine tumour necrosis factor-α^47^. It would thus be interesting, to uncouple MAP3K8 function in epithelial cancer cells and in immune cells to have a clear answer about MAP3K8 function in tumour development, considering both cell-autonomous and non-cell-autonomous functions. Interestingly, our study responds in part to that question. Indeed, by analysing cohorts of HGSC patients, we observed that MAP3K8 protein accumulation is associated with poor prognosis in HGSC patients. This further argues that the cell-autonomous function of MAP3K8 in ovarian cancers could be dominant over the anti-tumour effect mediated by CD8+ cytotoxic-T lymphocytes or by tumour necrosis factor-α-producing M1 macrophages. Thus, by combining analyses of HGSC patients, ovarian cancer cells and mouse xenograft models, we have unambiguously demonstrated that MAP3K8 fulfills a pro-tumourigenic role in ovarian cancers.

MAPK signalling leading to MEK/ERK activation has a central role in regulating the growth and survival of cells in many cancers, therefore it has long been perceived as attractive for new cancer therapies^65^. MEK is a direct substrate for MAP3 kinases, including BRAF and MAP3K8/TPL-2/COT. *MAP3K8* mutation is extremely rare in cancer^27^,^28^,^36^,^37^,^38^, unlike *BRAF* or its upstream regulator *KRAS*, in which somatic mutations occur with particularly high incidence in melanoma and lung cancers, respectively^35^,^66^. In human lung cancers, MAP3K8 is downregulated^64^, similar to what has been observed in melanoma with *BRAF* mutation^17^, possibly to prevent further MEK/ERK activation as a consequence of oncogenic *KRAS*/*BRAF* mutations. Moreover, some patients suffering from *BRAF*-mutated melanoma and treated with anti-BRAF compounds exhibit resistance to the treatment by reactivation of the MEK/ERK pathway through MAP3K8/TPL-2/COT accumulation^14^,^17^. Similarly, 70% cases of low-grade ovarian carcinomas harbour *KRAS* or *BRAF* mutations, leading invariably to constitutive activation of MEK/ERK^59^, while less than 1% of HGSC exhibit such mutations^4^,^12^. Still, half HGSC exhibit constitutive MEK/ERK activation, due to accumulation of MAP3K8/TPL-2/COT, the other MEK kinase besides BRAF. Thus, HGSC with high MAP3K8 protein levels mimic the pathological situation observed in melanoma, which become resistant to anti-BRAF therapy. In HGSC, MAP3K8 exhibits a reliable predictive value for the efficiency of MEK inhibitors. There are a small number of on-going clinical trials testing the potential benefit of MEK inhibitors in the treatment of ovarian cancers, but they are restricted to low-grade tumours because of their mutational status^60^. However, the objective response to MEK inhibitor treatment did not correlate with *BRAF* or *KRAS* mutational status, suggesting that other molecular markers might be involved in predicting sensitivity to such treatment^15^. According to our data, MAP3K8 could be such a biomarker. It would thus be interesting to test whether MAP3K8 could be a general prognostic marker in ovarian cancers irrespective of the grade. Thus, if anti-MEK therapies have been included in clinical trials for low-grade ovarian carcinoma, our results demonstrate that high-grade ovarian carcinoma patients might also benefit from anti-MEK treatments, combined with conventional chemotherapies.

## Methods

### Cohorts of HGSC patients

High-grade epithelial ovarian tumours of the serous histological subtype were collected from a cohort of 139 patients treated at the Institut Curie between 1989 and 2012, based on tumour grade, histological subtype and the availability of patient characteristics and clinical features that have been described in Supplementary Table 1 and its corresponding legend. The projects developed here are based on surgical residual tumour tissues available after histopathological analyses that are not needed for diagnostic purposes. There is no interference with the clinical practice. Analysis of tumour samples was performed according to the relevant national law on the protection of people taking part in biomedical research. All patients included in our study were informed by their referring oncologist that their biological samples could be used for research purposes and they gave their verbal informed consent. In case of patient refusal, which could be either orally expressed or written, residual tumour samples were not included in our study. The Institutional Review Board and Ethics committee of the Institut Curie Hospital Group approved all analyses realized in this study. Clinical characteristics of the TCGA cohort of patients have been previously described^4^,^7^, and freely available biological data have been downloaded from the following portal: http://cancergenome.nih.gov/.

### Immunohistochemical staining from HGSC and cell lines

For IHC on human HGSC, sections of paraffin-embedded tissues (3 μm) were stained using a streptavidin-peroxidase protocol and the Lab Vision Autostainer 480 (Thermoscienific) as previously described in refs 3, 67, 68. In brief, paraffin-embedded sections were incubated with specific antibodies recognizing MAP3K8 (1:200; SantaCruz #sc-720) or rabbit IgG antibody (1:1,500; Abcam #171870) in PBS solution at pH 7.6 containing 0.05% Tween 20 for 1 h, following unmasking in 1 × Tris/EDTA buffer, pH 9 (Dako#S2367) for 20 min at 97 °C. For quantification, two different investigators, including one pathologist, blindly evaluated at least five distinct areas of each tumour. Hscores of MAP3K8 staining in epithelial cells were given as a function of the percentage of positive epithelial cells multiplied by the staining intensity (ranging from 0 to 4). MAP3K8 Hscores in epithelial cells have been evaluated in tumour sections from 139 HGSC patients.

We validated the specificity of MAP3K8 antibody using SKOV3 cells in which MAP3K8 has been stably inactivated (see below MAP3K8-silenced cell lines and culture conditions). In brief, 20 × 10^6^ SKOV3 stable cell lines (shCtrl, shMAP3K8_1 and shMAP3K8_2) were plated into 15-cm Petri dishes. At 24 h post plating, cells were washed with room-temperature PBS, trypsinized and pelleted in PBS before fixation using alcohol, formalin and acetic acid (AFA) fixative followed by paraffin embedding. Sections of AFA-fixed paraffin-embedded cell lines (3 μm) were stained using the same protocol as this one described above on human HGSC samples. For staining quantification, at least six distinct areas of each cell pellet were evaluated by two different investigators. A Hscore was given as a function of the percentage of positive cells and the staining intensity from 0 to 4.

### Protein extracts and western blot analysis from HGSC samples

Among the human 139 HGSC samples analysed by IHC, we had access to 108 frozen tissues, from which we have extracted proteins to perform western blots analyses. Proteins from human HGSC tumours enriched in epithelial cancer cells (up to 73% in average, with a minimum percentage of 55%) were extracted using boiling lysis buffer (50 mM Tris pH 6.8, 2% SDS, 5% glycerol, 2 mM dithiothreitol, 2.5 mM EDTA, 2.5 mM EGTA, 4 mM Na3VO4 and 20 mM NaF) supplemented with 2 × Halt phosphatase inhibitor (Perbio #78420) and a complete EDTA-free protease inhibitor cocktail tablet (Roche #1836170). The protein extract was snap-frozen in liquid nitrogen and stored at −80 °C. Protein concentration was evaluated using the BCA Protein Assay kit—Reducing Agent Compatible according to the manufacturer's instructions (Thermo scientific). For western blot analysis, 20 μg proteins extracted from HGSC were loaded onto homemade 10% or precast 4–12% polyacrylamide gels (Invitrogen). After electrophoresis, the proteins were transferred to a 0.45-μM polyvinylidene difluoride transfer membrane (Immobilon-P, Millipore). Membranes were then blotted overnight at 4 °C with the appropriate primary antibodies: MAP3K8 (1:1,000; SantaCruz #sc-720), ABIN-2 (1:1,000; SantaCruz #sc-50526), actin (1:10,000; Sigma #A5441) or all the following antibodies from Cell Signalling Technology, phospho-MEK (1:1,000; #9121), MEK (1:2,000; #9126), phospho-ERK (1:1,000; #9106), ERK (1:2,000; #9102) and NF-κB1 p105 (1:1,000; #4717). Specific binding of antibodies was detected using appropriate peroxidase-conjugated secondary antibodies (Jackson ImmunoResearch Laboratories #115-035-003 and 115-035-045), and was visualized by enhanced chemiluminescence detection (Western Lightning Plus-ECL, PerkinElmer). Densitometry analyses of immunoblots were performed using ImageJ software.

### Human and mouse cell lines and cell culture conditions

For our study, we have chosen the human SKOV3 (from ATCC) and IGROV-1 (a kind gift from D. Lallemand and J.S. Brugge) epithelial ovarian cancer cell lines based on MAP3K8 protein levels, *TP53* status, histological subtype and capacity to grow in nude mice. The histological subtype of these cell lines has been previously reported^41^,^42^ and defined as serous for SKOV3 and mixed serous for IGROV-1. MAP3K8 protein levels in these cell lines have been determined by IHC and western blot analyses. SKOV3 and IGROV-1 have been tested for absence of mycoplasma contamination and were propagated in DMEM (Gibco) supplemented with 10% fetal bovine serum (FBS, PAA), penicillin (100 U ml^−1^) and streptomycin (100 μg ml^−1^) (Gibco). MOSECs were a kind gift from Dr K. Roby and P. Terranova (University of Kansas)^46^. Briefly, cells were obtained by gentle trypsinization of mouse ovaries. After repeated passages *in vitro*, cells undergo spontaneous transformation and become tumourigenic. MOSEC were propagated in DMEM (Gibco # 41966-029) supplemented with 4% FBS, penicillin (100 U ml^−1^), streptomycin (100 μg ml^−1^), insulin (5 μg ml^−1^), transferrin (5 μg ml^−1^) and sodium selenite (5 ng ml^−1^) (ITS mix, Sigma-Aldrich #I-1884).

### MAP3K8-silenced cell lines and culture conditions

For generation of MAP3K8-silenced stable cell lines derived from SKOV3 and IGROV-1, PLKO.1-derived vectors with two different shRNAs targeting human MAP3K8 (TRCN0000010012 and TRCN0000010013 for shMAP3K8_1 and shMAP3K8_2, respectively), or expressing a scrambled shRNA (shCtrl), were purchased from Sigma-Aldrich. Viruses were produced by co-transfection (with Lipofectamine 2000, Invitrogen) of 293T cells with the vector plasmid, a vesicular stomatitis virus envelope expression plasmid (Vsvg) and a second-generation packaging plasmid (pPax2). Purified viral particles were used at multiplicity of infection 5 to infect SKOV3 and IGROV-1 cells overnight. Infected cells were selected with puromycin (1 μg ml^−1^) (Gibco# A11138-03) for 1 week, before experimental use. Stable cell lines were propagated in DMEM (Gibco) supplemented with 10% FBS (PAA), penicillin (100 U ml^−1^), streptomycin (100 μg ml^−1^) (Gibco) and 1 μg ml^−1^ of puromycin (Gibco# A11138-03).

For the short interfering RNA (siRNA) experiment, 3 × 10^5^ SKOV3 cells were plated in six-well plates. After 24 h, cells were transfected with 20 nM of ON-Target plus non-targeting siRNA (siCtrl, Thermoscientific #D-001810-01-20), human ON-Target plus MAP3K8 siRNA (siMAP3K8, target sequence 5′- GCCAAGAGGUACCAUGGUU -3′, Thermoscientific #J-003511-16), or human ON-Target plus SMART pool p90RSK siRNA (siRSK, target sequence 5′- GUGGGCACCUGUAUGCUAU -3′, Thermoscientific #J-003025-13) using Dharmafect 1 transfection reagent according to the manufacturer's instructions (Thermoscientific #T-2001-02).

### Treatment with MAP3K8 inhibitor

SKOV3, IGROV-1 and MOSEC cell lines were plated in medium containing 10% FBS, and immediately treated with 10 μM MAP3K8 kinase inhibitor (KI, Calbiochem #616373) or the same volume of dimethylsulphoxide (DMSO) (KI vehicle medium) for the indicated times (see also below additive information for each experimental procedure). For signalling pathway analyses, cells were serum-starved overnight before KI treatment during 1 h, followed by serum stimulation at the indicated time points.

### Growth kinetics and cell viability and cell-cycle analysis

The number of living cells was measured by trypan blue exclusion using Vi-Cell analyzer (Beckman Coulter). Cell-cycle distributions were performed on ethanol-fixed cells, stained with propidium iodide and analysed by flow cytometry. Flow cytometry data were acquired using CellQuest Pro (Becton Dickinson) software on the FACS LSRII machine (Becton Dickinson) and were analysed using ModfitLT (Verity) software.

### Migration and invasion assays

Twelve-well cell culture insert, and 24-well Transwell BioCoat growth factor-reduced Matrigel invasion chambers (BD Biosciences, 8 μM pore size) were used for migration and invasion assays, respectively. After overnight serum starvation, cells were plated to the upper side of the Transwell device, in triplicates, in serum-free medium, whereas the lower well contained regular 10% FBS culture medium to create an FBS gradient. We plated 80,000 cells for the migration assay and ended the experiment after 5 or 14 h for SKOV3 and IGROV-1, respectively (according to their respective migratory capacities). For the invasion assay, we plated 50,000 cells and stopped the experiment after 24 or 48 h for SKOV3 and IGROV-1, respectively (here again, defined according to their respective migratory capacities). At the end of the experiment, the remaining cells in the upper side of the Transwell device were removed. Migrating and invading cells at the bottom side of the Transwell device were fixed and stained with crystal violet for 30 min and then counted in five different representative fields (× 5 objective, Zeiss Axioplan microscope, AxioCamERc 5s).

### Protein extracts and western blot analysis from cell lines

SKOV3 and IGROV-1 underwent overnight serum starvation and were treated with 10 μM MAP3K8 kinase inhibitor (KI, Calbiochem #616373) or the same volume of DMSO (KI's vehicle) for 1 h before 10% FBS stimulation for the indicated times. Similarly, after overnight serum starvation, stable cell lines (shCtrl, shMAP3K8_1&2) derived from SKOV3 and IGROV-1 were stimulated with 10%FBS for 1 h or 15 min, respectively, based on the maximum of P-MEK activation shown on Fig. 3a. After treatment, cells were washed once with cold PBS and scraped on ice with lysis buffer (50 mM Tris-HCl pH 7.5, 1 mM EDTA, 1 mM EGTA, 1% triton X-100, 1 mM Na3VO4, 50 mM NaF, 0.27 M sucrose and 0,1% β-mercaptoethanol) supplemented with EDTA-free protease inhibitor cocktail tablet (Roche #1836170). Protein concentrations were evaluated using a Bio-Rad D/C protein assay. For western blot analysis, 10 μg proteins were loaded onto homemade 10% or precast 4–12% polyacrylamide gels (Invitrogen). After electrophoresis, the proteins were transferred to a 0.45-μM polyvinylidene difluoride transfer membrane (Immobilon-P, Millipore). Membranes were then blotted overnight at 4 °C with the appropriate primary antibodies: MAP3K8 (1:1,000; SantaCruz #sc-720), Phospho-MAP3K8 (T290) (1:1,000; Invitrogen #441370), P-FAK (Ser910) (1:1,000; Invitrogen #44596), p53 clone DO-1 (1:500; Thermoscientific #MS-187-PO), actin (1:1,000; Sigma #A5441) and Myc (9E10) (1:1,000; Roche #11667149001) or all the following antibodies from Cell Signalling Technology, Phospho-MAP3K8 (S400) (1:1,000; #4491), Phospho-MEK (1:1,000; #9121), MEK (1:2,000; #9126), Phospho-ERK (1:1,000; #9106), ERK (1:2,000; #91022), phospho-JNK (1:1,000; #4668), JNK (1:1,000; #9258), phospho-NF-κB p65 (1:2,000; #3033), NF-κB p65 (1:2,000; #8242), phospho-p38 (1:1,000; #4511), p38 (1:2,000; #9218), phospho-p90RSK (Ser380) (1:1,000; #9335), p90RSK (1:2,000; #8408), FAK (1:2,000; #3285) and cyclin D1 (1:500; #2922). Specific binding of antibodies was detected using appropriate peroxidase-conjugated secondary antibodies (Jackson ImmunoResearch Laboratories #115-035-003 and 115-035-045), and was visualized by enhanced chemiluminescence detection (Western Lightning Plus-ECL, PerkinElmer). Densitometric analyses of immunoblots were performed using ImageJ software.

### Immunoprecipitation

For immunoprecipitation (IP), 4 × 10^6^ SKOV3 cells were plated into a 10-cm Petri dish. After 6 h, cells were transfected with increasing amounts (2–6 μg) of N-terminal myc-tagged human MAP3K8 pcDNA3.1(+) plasmid DNA (Source BioScience imaGenes) using JetPrime transfection reagent according to the manufacturer's instructions with 1:4 ratio (Polyplus transfection) or with empty vector (Ctrl). At 48 h post transfection, cells were washed with cold PBS and scraped on ice with IP lysis buffer (50 mM Tris-HCl pH 7.5, 150 mM NaCl, 1 mM EDTA, 1 mM EGTA, 1% triton X-100, 1 mM Na3VO4, 10 mM β-glycerophosphate, 50 mM NaF, 5 mM sodium pyrophosphate, 0.27 M sucrose and 0.1% β-mercaptoethanol) supplemented with a EDTA-free protease inhibitor cocktail tablet (Roche #1836170). Cell extracts were centrifuged at 13,000 r.p.m. for 10 min at 4 °C. The protein concentration of the supernatant was determined using the Bio-Rad Dc Protein Assay Kit according to the manufacturer's instructions (Bio-Rad Laboratories). After 10 min incubation on ice, protein lysates were spun down at 13,000 r.p.m. and the supernatants were transferred into a fresh tube. For IP, 500 μg of protein extract were processed immediately and incubated on a wheel overnight at 4 °C, with 33 μl of myc antibody (9E10) coupled to magnetic beads (Dynabeads antibody coupling kit #1143.11D, Invitrogen) at 30 μg antibody per milligram dynabeads. Beads were then washed three times using IP lysis buffer. Finally, 20 μl of samples buffer 2 × were added on top of the beads and boiled for 5 min at 95 °C. Western blot analysis of IP samples were then performed as described above.

### Xenograft experiments

PDX models of HGSC tumours were established at the Institut Curie (Paris, France) with patient consent. Tumour fragments of 30–60 mm^3^ were grafted into the interscapular fat pad of 6-week-old female Swiss nude mice, under avertin anaesthesia. Xenograft appeared at the graft site 2–6 months after grafting. At least eight PDX mice per group were treated *per os* five times a week for 4 weeks with MEK inhibitors either AZD6244 at 50 mg kg^−1^ (Selleckchem #S100817) or MEK162 25 mg kg^−1^ (Active Biochem #A-1128) both diluted into 2% DMSO, 10% 2-hydroxypropyl-beta-cyclodextrin and 5% glucose in PBS. For combination of MEK inhibitor with conventional chemotherapy, at least eight PDX mice per group were treated once every 3 weeks for 6 weeks with intraperitoneal injection of carboplatine at 33 mg kg^−1^ (Onco-tain) diluted in H_2_O and paclitaxel at 15 mg kg^−1^ (Fresenius Kabi) diluted in 0.9% NaCl. Conventional chemotherapy was either administrated alone or in combination with MEK162 (as described above) for 6 weeks. Tumour growth was evaluated by measuring two perpendicular diameters of tumours with a caliper twice a week. Individual tumour volumes were calculated as (*V)*=*a* × *b*^2^/2, with *a* being the major and *b* the minor diameter. For each tumour, the tumour volume at day *n* (*V*_n_) was reported as the initial volume before inclusion in the experiment (*V*_0_) and expressed as relative tumour volume (RTV) according to the following formula: RTV=*V*_*n*_/*V*_0_. Means and s.e.m. of RTV in the same treatment group were calculated, and growth curves were established as a function of time. The tumour growth inhibition was calculated using the following formula: 100−100 × (RTV from control mice/RTV from MEK inhibitor-treated mice).

Grafting experiments were performed by subcutaneous injection of 2.7 × 10^6^ exponentially growing SKOV3- and IGROV-1-derived stable cell lines shCtrl, shMAP3K8_1 and -2 into each flank of 6-week-old female Swiss nude mice (at least five mice per group). Tumour growth was evaluated twice a week, as mentioned above, for 30–60 days.

All protocols involving mice and animal housing were in accordance with institutional guidelines as proposed by the French Ethics Committee and have been approved (agreement number: CEEA-IC #118: 2013-06).

### qRT–PCR

Human RNAs were analysed for *MAP3K8* expression using real-time reverse transcription–PCR (RT–PCR). The RT–PCR protocol using the SYBR Green Master Mix kit on the ABI Prism 7900 Sequence Detection System (PerkinElmer Applied Biosystems, Foster City, CA) has been described previously^68^. The primer pair used for quantitative (q)RT–PCR amplification of *MAP3K8* gene (forward and reverse primers) was: 5′- GGCCGCAGATGCAATCTTCTTA -3′ and 5′- TGGCTTTGCAGATACTGCGTT -3′. *MAP3K8* mRNA levels were determined as the mean of the Ct values obtained from the couple of primers. We also quantified the *GAPDH* (glyceraldehyde-3-phosphate dehydrogenase) mRNA level, as an endogenous RNA control for the total amount of RNA in each tumour sample. We normalized *MAP3K8* mRNA levels on the basis of the expression level of *GAPDH*. The primer pair used for quantitative RT–PCR amplification of *GAPDH* gene (forward and reverse primers) was: 5′- CTTCAACAGCGACACCCACT -3′ and 5′- GTGGTCCAGGGGTCTTACTC -3′. Total RNA extraction, complementary DNA synthesis and PCR reaction conditions have been described previously^68^. Results, expressed as relative *MAP3K8* gene expression were determined as relative *MAP3K8*=2^−(Ct *MAP3K8—*Ct *GAPDH*)^.

For lung metastasis detection in patient-derived tumour xenografts, the real-time PCR allows the detection of human RNA from mouse tissues. The presence of human cells within the host organ was quantified by mean of the transcript of human genes highly and exclusively represented in the human genome (Alu sequences). This method applied to the xenograft models greatly enhances the sensitivity of detection of invading human cells within lung tissues and has been described in ref. 68. Alu transcripts were considered to be detectable and quantifiable when the Ct value was below 35, and not detectable when the Ct value was above 35. The primers used for detection of Alu sequence were the following: 5′- TCACACCTGTAATCCCAGCACTTT -3′ and 5′- GCCCAGGCTGGAGTGCAGT -3′.

### Statistical analysis

To evaluate the prognostic value of MAP3K8 protein, we used the MAP3K8 Hscore determined by IHC analysis of MAP3K8 staining in HGSC tumours, and subdivided the HGSC patients into two groups defined as low or high MAP3K8 based on the median. The Kaplan–Meier curves were compared using the log-rank test. For all statistical analysis, differences were considered to be statistically significant at values of *P*≤0.05. Graphs generally represent mean±s.e.m. obtained from independent experiments using an adapted statistical test, as mentioned in the legends of the figures. The horizontal dark line on the scatter plots represents the mean, and the error bars the s.e.m. Spearman's correlation test was used to evaluate the correlation coefficient between two parameters. All statistical analyses were performed using R or Prism software.

## Additional information

**How to cite this article:** Gruosso, T. *et al*. MAP3K8/TPL-2/COT is a potential predictive marker for MEK inhibitor treatment in high-grade serous ovarian carcinomas. *Nat. Commun.* 6:8583 doi: 10.1038/ncomms9583 (2015).

## Supplementary Material

Supplementary Information Supplementary Figures 1-8 and Supplementary Tables 1
