# Supplementary material for: MAP3K8/TPL-2/COT is a potential predictive marker for MEK inhibitor treatment in high-grade serous ovarian carcinomas
Source: Nat Commun. 2015 Oct 12;6:8583. doi: 10.1038/ncomms9583 (PMC4633961; doi:10.1038/ncomms9583)
Supplement: Supplementary Information — Supplementary Figures 1-8 and Supplementary Tables 1 [file ncomms9583-s1.pdf]

SUPPLEMENTARY INFORMATION

MAP3K8/TPL-2/COT is a potential predictive marker for MEK inhibitor treatment in high-grade serous ovarian carcinomas

Tina Gruosso[1,2] , Camille Garnier[1,2], Sophie Abelanet[1,2], Yann Kieffer[1,2], Vincent Lemesre[1,2], Dorine Bellanger[3,2],Ivan Bieche[4], Elisabetta Marangoni[5], Xavier Sastre-Garau[6], Virginie Mieulet[1,2, §,] * and Fatima Mechta-Grigoriou[1,2, §,] *

[1] Stress and Cancer Laboratory, Institut Curie, 26, rue d'Ulm, 75248 Paris, France

[2] Inserm, U830, Paris, F-75248, France

[3] Genomics and Biology of the Hereditary Breast Cancers, Institut Curie, 26, rue d'Ulm, 75248 Paris, France

[4] Department of Pharmacogenomics, Institut Curie, 26, rue d'Ulm, 75248 Paris, France

[5] Laboratory of Precinical Investigation, Translational Research Department, Institut Curie, 26, rue d'Ulm, 75248 Paris, France

[6]Department of Pathology, Institut Curie, 26, rue d'Ulm, 75248 Paris, France

Running title: MAP3K8/TPL-2/COT functions in ovarian tumourigenesis

Keywords: MAP3K8, COT, TPL-2, high-grade, epithelial ovarian cancers, MAPK, MEK inhibitors

[§] These authors equally contributed to the work

* Co-corresponding authors

Correspondence and requests for materials should be addressed to V.M. (email: virginie.mieulet@curie.fr) and F.M.G. (email: fatima.mechta-grigoriou@curie.fr)

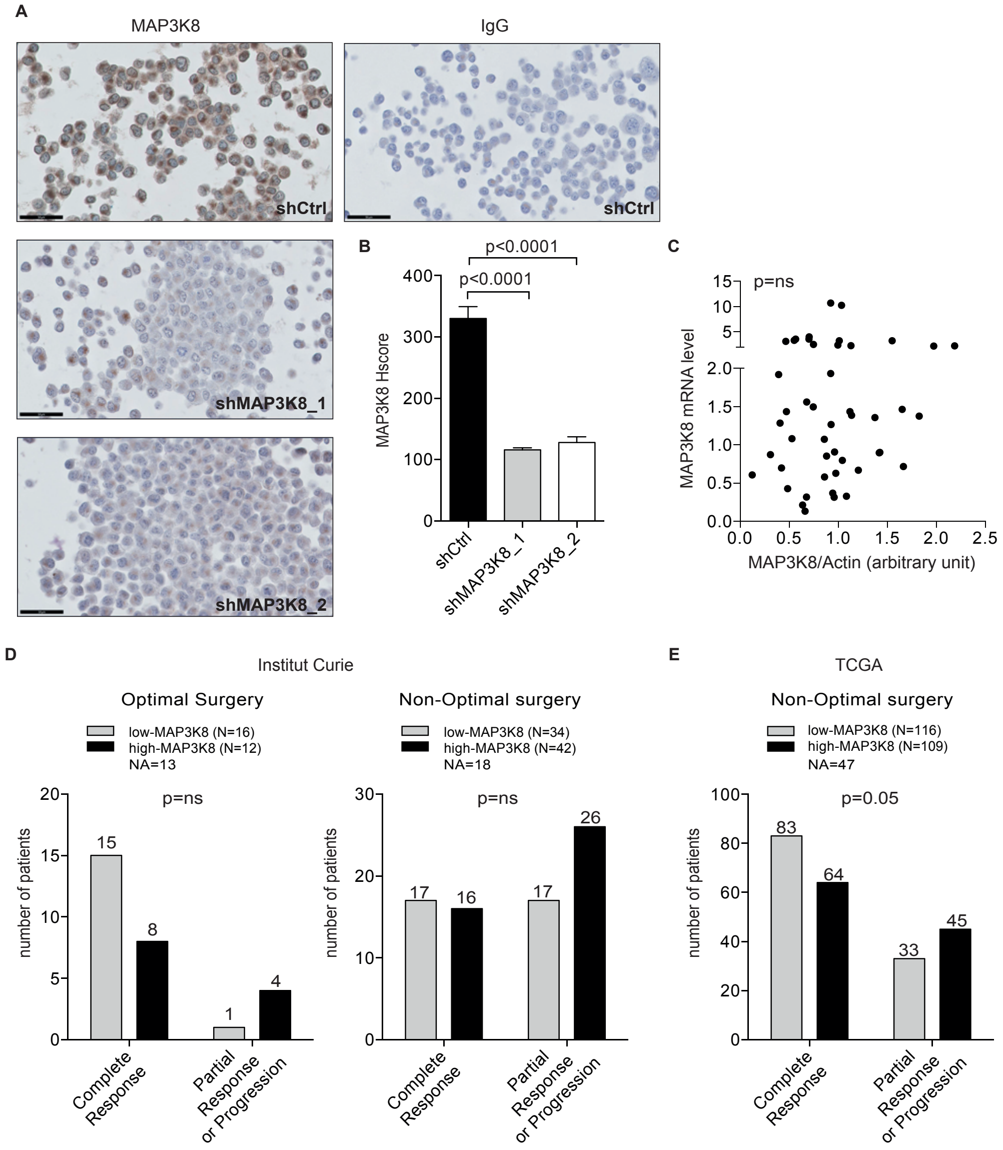

Supplementary Figure 1: Specificity of MAP3K8 antibody

(A) Representative views of MAP3K8 immunostaining from SKOV3 cell lines stably expressing non-targeting shRNA (shCtrl) or 2 different MAP3K8-targeting shRNA (shMAP3K8_1 and shMAP3K8_2), as indicated. On the right is shown a representative view of immunostaining from SKOV3 obtained with a rabbit IgG antibody, used as a matched isotype control for MAP3K8 staining. Scale bars: 50 µm. (B) Bar plots of MAP3K8 histological score (Hscore, see methods) determined by quantitative analysis of MAP3K8 immunostaining (IHC), as shown in (A). P-values are based on Student's t-test. Data are shown as means ± s.e.m. (C) No significant correlation between MAP3K8 protein levels and mRNA levels in human HGSC. MAP3K8 protein levels have been assessed by densitometry analysis of western blots, as those shown in (Figure 4A), analysing MAP3K8 RNA and protein levels from the same HGSC patients. MAP3K8 mRNA levels have been assessed by qRT-PCR analysis of human HGSC samples. Correlation coefficient σ and p-value are based on Spearman's rank correlation test. ns, not significant. (D) Bar-plots testing association of MAP3K8 protein level with clinical response in fully resected tumours (Optimal surgery, Left) and partially resected tumours (Non-optimal surgery, Right). Data are from Institut Curie cohort of patients, only HGSC of advanced stage (Stage III or IV) are considered. The numbers of patients for whom we had access to all required information (grade, stage, debulking status, MAP3K8 protein level and response to treatment) are indicated above each plot bar. (E) Similar analysis as in (D) showing the association between the phosphorylated form of MEK and the response to treatment. Data are from the TCGA cohort of patient. Only patients with HGSC of advanced stage (Stage III or IV), who experienced a partial debulking, are considered. P-values are based on Fisher's Exact test.

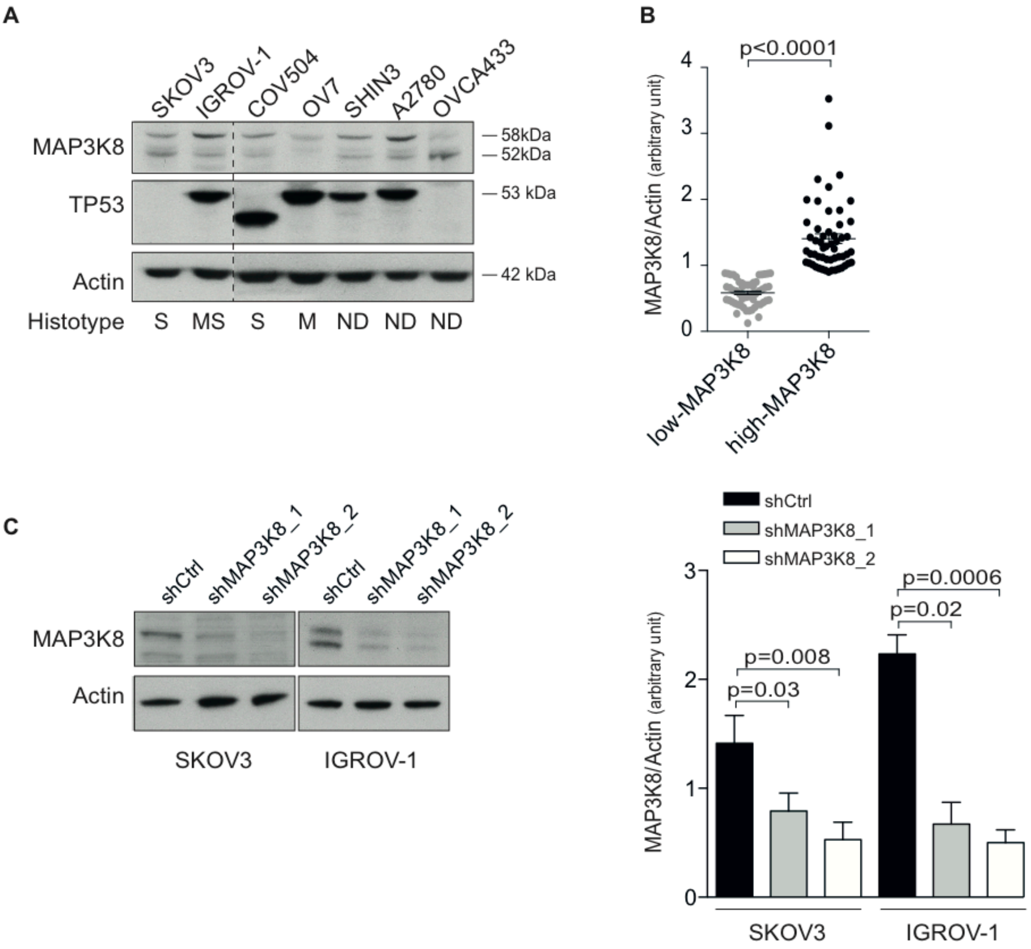

Supplementary Figure 2: MAP3K8 protein levels in ovarian cancer cell lines

(A) Representative western blots showing MAP3K8 and TP53 protein levels in a set of ovarian cancer cell lines including SKOV3, IGROV-1, COV504, OV7, SHIN3, A2780 and OVCA433, as indicated. Actin is used as an internal control for protein loading. The histological subtype, when identified for these cell lines in previous studies [40,41], is also indicated as: S=Serous, MS= Mixed Serous, M=Mixed, ND: not determined. SKOV3 (*TP53* null) and IGROV-1 (*TP53* mutated), of serous histotype, exhibit high levels of MAP3K8 protein and have been used for further analyses. (B) Scatter plot of MAP3K8 protein levels in 108 HGSC tumour samples, as assessed by densitometry analyses of western blots (as shown in Figure 4A). Two subgroups of HGSC patients have been defined, as low-MAP3K8 (N=54 patients) or high-MAP3K8 (N=54 patients) based on the median (=0.9). P-value is based on Student's t-test. (C) Left: Representative western blot showing MAP3K8 protein levels in stable cell lines (shCtrl, shMAP3K8_1 and shMAP3K8_2) derived from SKOV3 and IGROV-1. Actin is used as an internal control for protein loading. Right: Bar plots of MAP3K8 protein levels in stable cell lines (shCtrl, shMAP3K8_1 and shMAP3K8_2), assessed by densitometry analyses of western blots, as shown in Left (n=3 independent experiments). P-values are based on Student's t-test. Data are shown as means ± s.e.m. The mean values obtained for parental SKOV3 and IGROV-1 cell lines (mean value =1.4 for SKOV3 and 2.2 for IGROV-1) are above 0.9, the median of MAP3K8 integrated density separating low- and high-MAP3K8 HGSC tumour samples (as shown in B). Moreover, the mean values obtained for shMAP3K8_1 and shMAP3K8_2 stable cell lines derived from SKOV3 and IGROV-1.

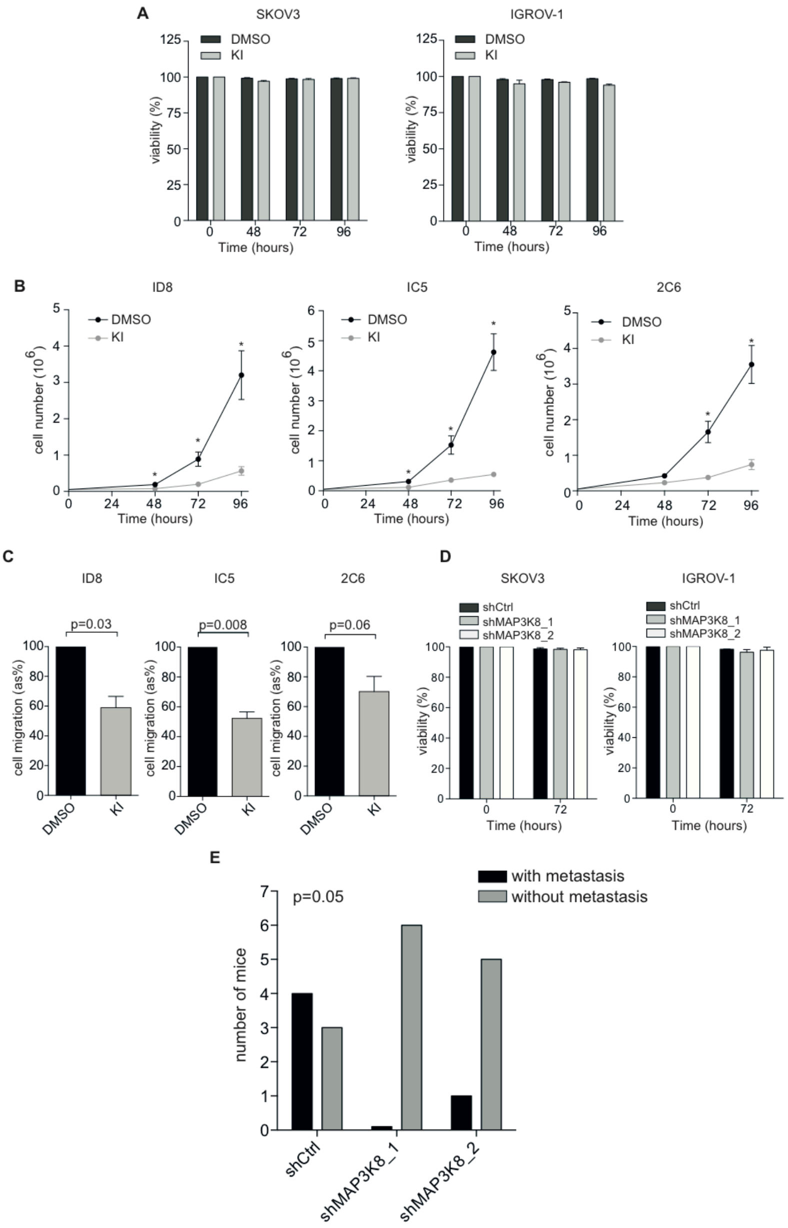

Supplementary Figure 3: MAP3K8 inhibition does not affect cell viability and MAP3K8 cell autonomous functions are conserved in murine ovarian cancer cell lines

(A) Bar plot showing cell viability of SKOV3 and IGROV-1 ovarian cancer cell lines either treated with MAP3K8 kinase inhibitor (KI) or with the vehicle medium for KI (DMSO). Cell viability was assessed by trypan blue exclusion and is expressed as a percentage of viable cells, at the indicated times. Data are shown as means ± s.e.m (n=3 independent experiments). (B) Growth curve of MOSEC (Mouse Ovarian Surface Epithelial Cells) cell lines ID8 (left panel), IC5 (middle panel) and 2C6 (right panel) either treated with MAP3K8 kinase inhibitor (KI) or with the vehicle medium for KI (DMSO), for the indicated times. P-values are based on Student’s t-test. * Stands for p-value≤0.05. Data are shown as means ± s.e.m (n=3 independent experiments). (C) Bar plots representing cell migration of MOSEC cell lines ID8 (left panel), IC5 (middle panel) and 2C6 (right panel) either treated with MAP3K8 kinase inhibitor (KI) or with the vehicle medium for KI (DMSO). P-values are based on one-sample t-test. Data are shown as means ± s.e.m (n=3 independent experiments). (D) Bar plot showing cell viability of stable cell lines (shCtrl, shMAP3K8_1 and shMAP3K8_2) derived from SKOV3 and IGROV-1 cancer cells. Cell viability was assessed by trypan blue exclusion and is expressed as a percentage of viable cells, at the indicated times. (E) The bar plot shows the number of mice without (grey) or with (black) lung metastases, as assessed by the detection of human-specific Alu sequences in RNA extracted from mouse lung samples. Mice were grafted with SKOV3 cells stably transfected with non-targeting shRNA (shCtrl) or with 2 different MAP3K8-targeting shRNA (siMAP3K8_1 and siMAP3K8_2), as indicated (N≥7 mice per condition). P-value is based on Fischer’s exact test.

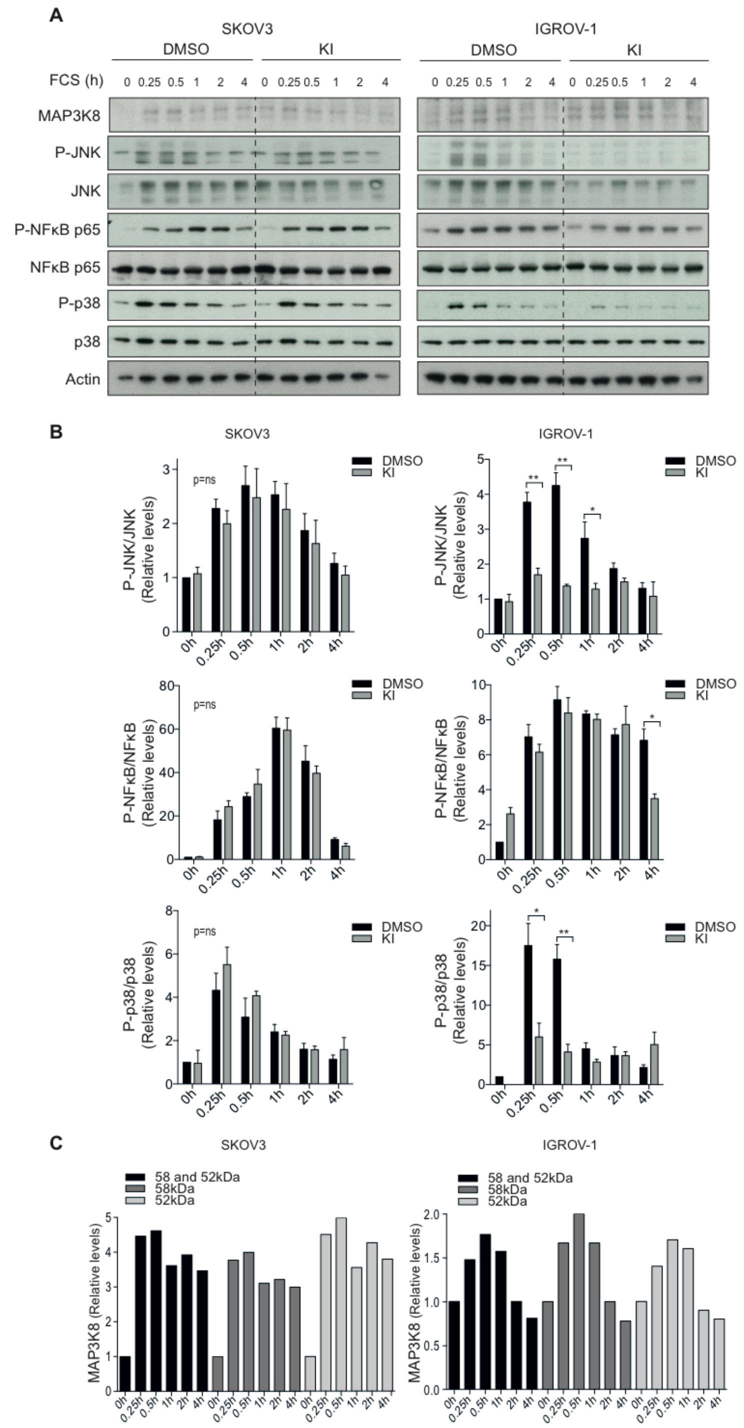

Supplementary Figure 4: MAP3K8 inhibition by KI treatment does not affect JNK, p38MAPK and NF-κB p65 pathway reproducibly in ovarian cancer cell lines

(A) Representative western blots showing MAP3K8, P-JNK, JNK, P-NFκB, NFκB, P-p38, and p38 protein levels upon serum (FBS) stimulation for the indicated times in SKOV3 and IGROV-1 ovarian cancer cell lines treated prior to FBS stimulation either with MAP3K8 kinase inhibitor (KI) or with the vehicle medium for KI (DMSO). Actin is used as an internal control for protein loading. (B) Bar plots showing P-JNK/JNK, P-NFκB/NFκB, P-p38/p38 ratios, as assessed by densitometry analysis of western blots (as shown in A) obtained from independent experiments and expressed as fold change compared to the DMSO t=0h time point. P-values are based on Student's t-test. Data are shown as means ± s.e.m (n=3 independent experiments). (C) Bar plots showing MAP3K8 protein levels following serum induction of SKOV3 and IGROV-1 ovarian cancer cells, as indicated. Are considered either both p58 (kDa) and p52 (kDa) MAP3K8 isoforms (black), or each isoform (p58, dark grey or p52, light grey), as assessed by densitometry analysis of western blots (as shown in A) and expressed as fold change compared to t=0h time point.

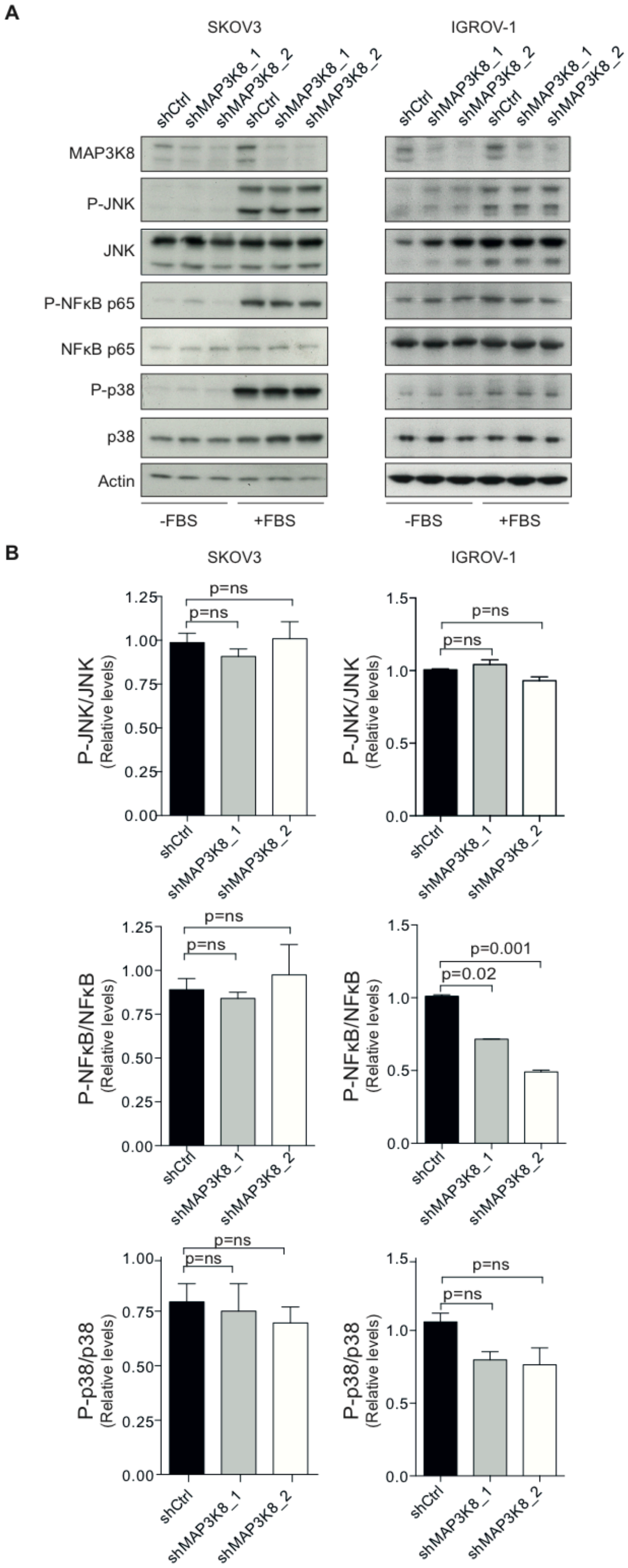

Supplementary Figure 5: MAP3K8 silencing using shRNA does not affect JNK, p38MAPK and NF-κB p65 pathway reproducibly in ovarian cancer cell lines

(A) Representative western blots showing MAP3K8, P-JNK, JNK, P-NF-κB, NF-κB, P-p38, and p38 protein levels in stable cell lines (shCtrl, shMAP3K8_1 and shMAP3K8_2) derived from SKOV3 and IGROV-1 cancer cells and kept without serum (-FBS) or stimulated by serum (+FBS). Actin is used as an internal control for protein loading. (B) Bar plots showing P-JNK/JNK, P-NF-κB/NF-κB, P-p38/p38 ratios, as assessed by densitometry analysis of western blots (as shown in A). P-values are based on Student's t-test. Data are shown as means ± s.e.m (n≥3 independent experiments).

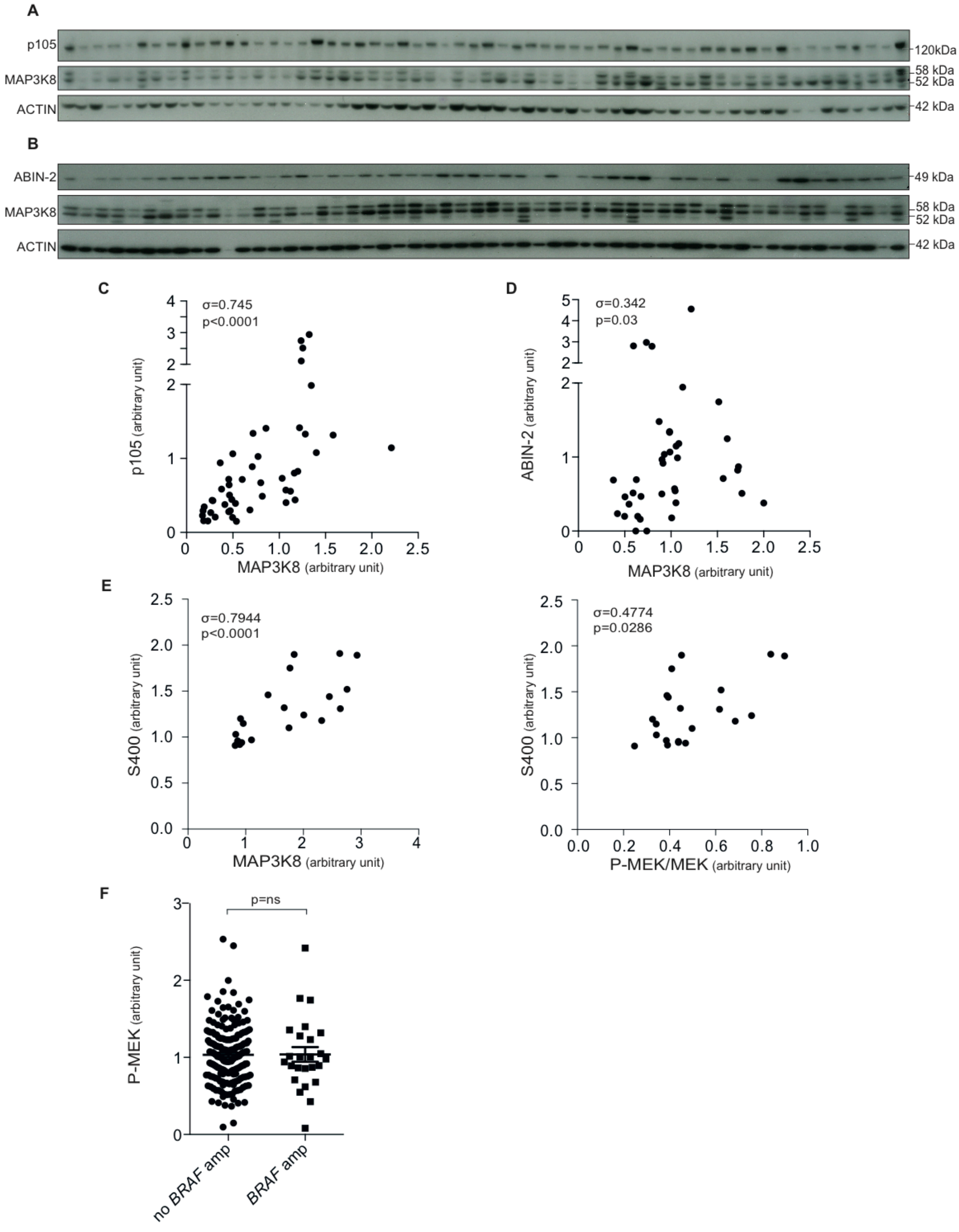

Supplementary Figure 6: MAP3K8 protein levels correlate with NF-κB1 p105, ABIN-2 and MEK activation

(A,B) Representative western blots showing MAP3K8, NF-κB1 p105 (A) or ABIN-2 (B) protein levels in human HGSC (N≥54 patients). Actin is used as an internal control for protein loading. (C,D) Correlation plots between MAP3K8 and NF-κB1 p105 (C) or ABIN-2 (D), in human HGSC. Values have been assessed by densitometry analysis of western blots (as those shown in A,B). Correlation coefficient σ and p-value are based on Spearman's rank correlation test. (E) Correlation between P-MAP3K8 (S400) and either MAP3K8 protein levels (left panel) or P-MEK/MEK ratio (right panel). Values have been assessed by densitometry analysis of western blots, as shown in Figure 4E. Correlation coefficient σ and p-value are based on Spearman's rank correlation test (n=4 independent experiments). (F) Scatter plot showing the level of P-MEK in human HGSC without amplification of the *BRAF* gene (no *BRAF* amp, N= 182 tumours) or exhibiting *BRAF* gene amplification (*BRAF* amp, N=25). P-value is based on Student's t-test. Data are shown as means ± s.e.m. (N=217 HGSC). Data are from the TCGA (http://cancergenome.nih.gov/)[4].

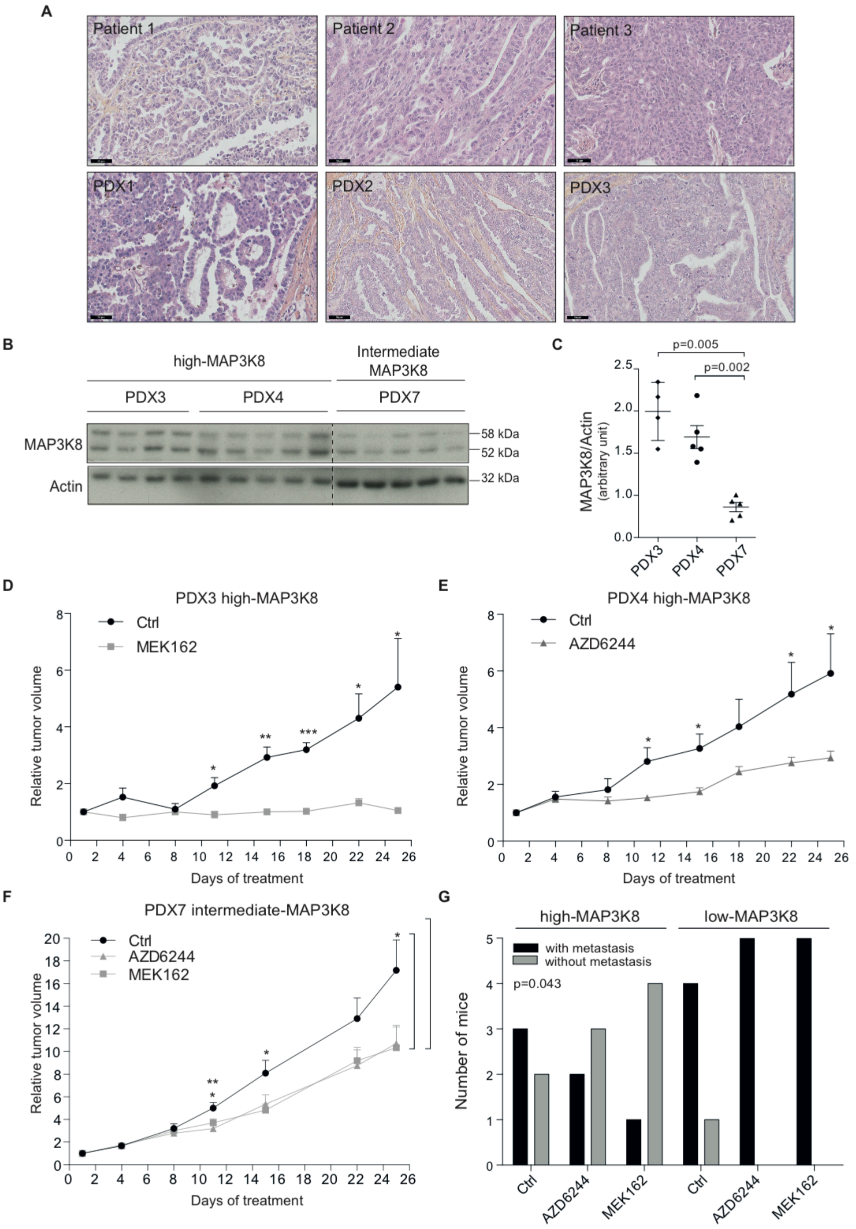

Supplementary Figure 7: MEK inhibitor treatment impairs tumour growth in high-MAP3K8 PDX models

(A) Representative views of HES staining from human primary HGSC tumours and the corresponding PDX tumours derived from these primary tumours. Three relevant PDX models used in our study (PDX1,PDX2,PDX3) are shown here and demonstrate that histological properties are conserved from the primary tumours to their corresponding PDX. Scale bars: 50 µm. (B) Left: Western blot showing MAP3K8 protein levels in at least 4 different tumours derived from PDX models with high- (PDX3,PDX4) or intermediate- (PDX7) MAP3K8 protein levels, relative to the low-MAP3K8 PDX models, as shown (Figure 5A). Actin is used as an internal control for protein loading. (C) Scatter plots of MAP3K8/Actin ratios in tumours from PDX3, PDX4 and PDX7 models, as assessed by densitometry analysis of the western blots, as shown in (B). P-values are based on Student's t-test. Data are shown as means ± s.e.m. (N≥4 tumours per group). (D-F) Relative tumour volume over time in PDX3 (D), PDX4 (E) and PDX7 (F). Mice were either untreated (Ctrl, black lines) or treated with MEK inhibitors, such as AZD6244 and MEK162 (grey lines), as indicated. P-values are based on Student's t-test. * stands for p-value≤0.05 and **, p-value≤0.005, *** stands for p-value≤0.0005. Data are shown as means ± s.e.m (N≥4 mice per group). (G) The number of mice without (grey) or with (black) lung metastases, as assessed by the detection of human-specific Alu sequences in RNA extracted from mouse lung samples. Mice from high- or low-MAP3K8 PDX models were either untreated (Ctrl) or treated with MEK inhibitors, AZD6244 or MEK162, as indicated (N=5 mice per condition). P-value is based on Fischer's exact test.

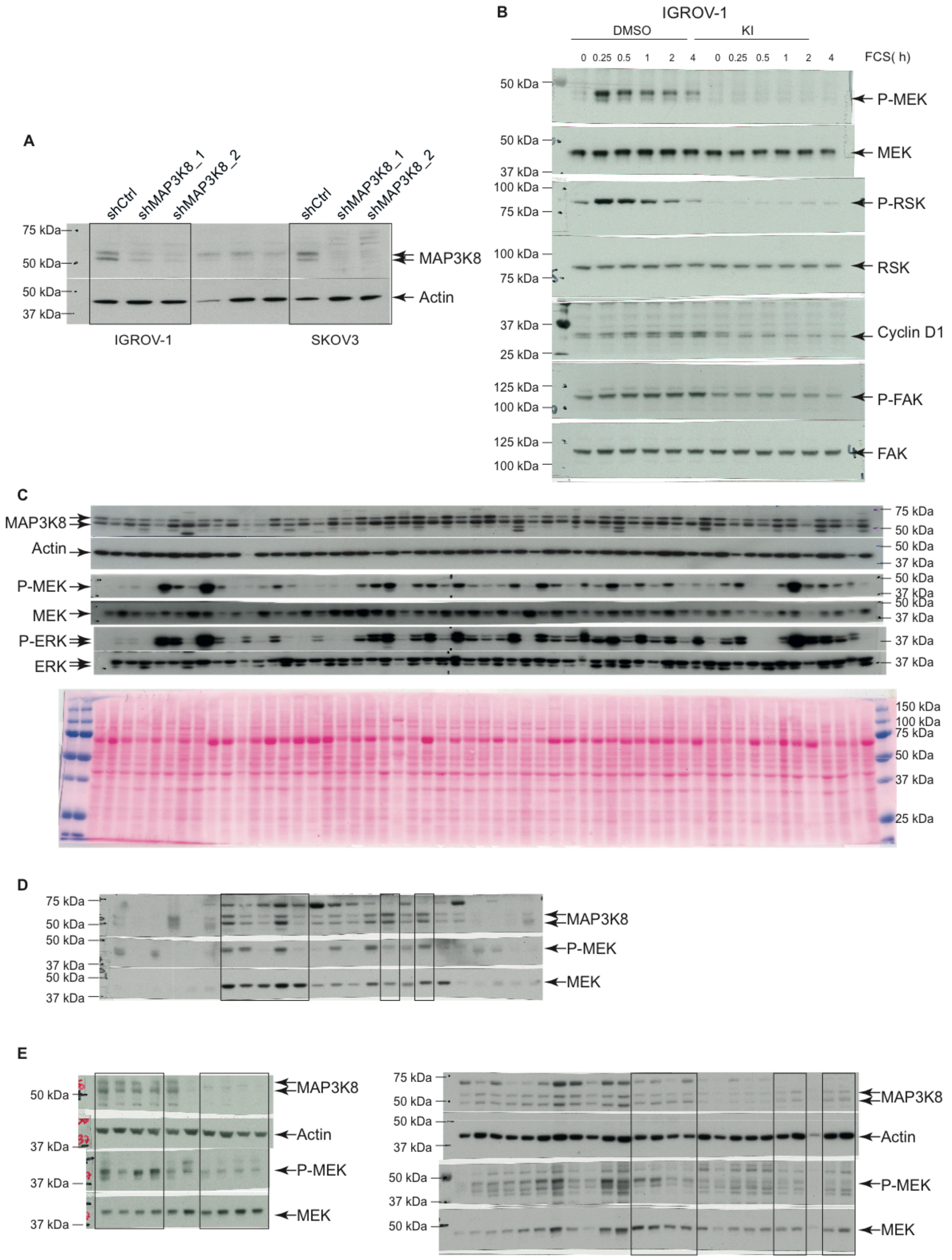

Supplementary Figure 8: Full western blots shown in main figures

(A) Portions of these western blots are shown in Figure 2D. (B) Full blots presented as one example of the full blots shown in Figures 3A,G. (C) Portions of these western blots are shown in Figure 4A. (D) Portions of these western blots are shown in Figure 5A. (E) Portions of these western blots are shown in Figure 5D. All the other blots shown in Figures 3C,3G, 4D and 4F are full blots.

Total number of tumors		
		139
Median age (range) - years		
	Median age	61 years
	Range	35-87 years
Histotype		
	Serous	139 (100%)
Figo substage		
	Ia	4 (2.9%)
	Ib	3 (2.2%)
	Ic	4 (2.9%)
	IIa	3 (2.2%)
	IIb	9 (6.5%)
	IIc	2 (1.4%)
	IIIa	4 (2.9%)
	IIIb	2 (1.4%)
	IIIc	91 (65.5%)
	IV	17 (12.2%)
Grade		
	High-grade	139 (100%)
Surgery		
	Optimal	41 (29.7%)
	Non-optimal	94 (67.6%)
	NA	4 (2.9%)
Chemotherapy		
	Yes	131 (94.2%)
	No	8 (5.8%)
Chemoterapeutic agents		
	Alkylating agents	25 (19%)
	Alkylating agents + taxanes	106 (81%)
Clinical response		
	Complete Response	56 (40.3%)
	Partial response/Progression	48 (34.5%)
	NA	35 (25.2%)
BRCAness		
	High-LST (BRCAness)	34 (53%)
	Low-LST (non-BRCAness)	30 (47%)
Relapse		
	Yes	115 (82.7%)
	No	24 (17.3%
Median delay of relapse (range) - months		
	Median delay of relapse	19.2
	Range	0.1-243

Supplementary Table 1: Main patient characteristics and clinicopathological features of HGSC in Institut Curie cohort

Tumour samples were obtained from a cohort of consecutive ovarian carcinoma patients, treated at the Curie Institute between 1989 and 2012. All analysed samples have been collected prior to any chemotherapeutic treatment. Indeed, for each patient, a surgical specimen was taken, before chemotherapy, for pathological analysis and tumour tissue cryopreservation. The median's patient's age was 61 years (with a range of 35-87 years). Ovarian carcinomas were classified according to the World Health Organization histological classification of gynaecological tumours. Pathological analysis identified 139 high-grade serous tumours (100%). 25 subjects (18%) were considered as early stage (International Federation of Gynaecology and Obstetrics (FIGO) I-II) and 114 subjects (82%) were considered as advanced stage (III and IV) of disease. Patients were treated with a combination of surgery and chemotherapy, the latter including alkylating or alkylating-like agents ± taxane as a first line treatment in most cases. Clinical response was evaluated according to tumour mass evolution by monitoring patients throughout the chemotherapeutic treatment. 56 subjects (40.3%) had a complete response to treatment and 48 subjects (34,5%) had a partial response or progression. Complete response was defined as complete disappearance of the disease up to 6 months after treatment. In patients with incomplete response, the disease does not significantly decrease or even progress during treatment, or shows recurrence within 6 months of treatment completion. All the subjects underwent surgery, 94 of them have a non-optimal surgery and 41 subjects have an optimal surgery. 115 patients (82.7%) relapsed with a median delay of relapse of 24 months (with a range of 0.1-243 months). Inactivation of genes associated with homologous recombination repair or BRCA1 methylation (BRCAness status) were defined by large-scale state transitions, referring to as chromosomal breaks between adjacent regions of at least 10 Mb, which are robust indicator of BRCAness status in ovarian and basal-like breast cancers [68].
